# Human cytomegalovirus-induced host protein citrullination is crucial for viral replication

**DOI:** 10.1038/s41467-021-24178-6

**Published:** 2021-06-23

**Authors:** Gloria Griffante, Francesca Gugliesi, Selina Pasquero, Valentina Dell’Oste, Matteo Biolatti, Ari J. Salinger, Santanu Mondal, Paul R. Thompson, Eranthie Weerapana, Robert J. Lebbink, Jasper A. Soppe, Thomas Stamminger, Virginie Girault, Andreas Pichlmair, Gábor Oroszlán, Donald M. Coen, Marco De Andrea, Santo Landolfo

**Affiliations:** 1 Department of Public Health and Pediatric Sciences, University of Turin, Turin, Italy; 2 Department of Translational Medicine, University of Piemonte Orientale, Novara, Italy; 3 Department of Biochemistry and Molecular Pharmacology, UMass Medical School, Worcester, MA USA; 4 Department of Chemistry, Boston College, Chestnut Hill, MA USA; 5 Department of Medical Microbiology, University Medical Center Utrecht, Utrecht, The Netherlands; 6 Institute of Virology, Ulm University Medical Center, Ulm, Germany; 7 Institute of Virology, Technical University of Munich, Munich, Germany; 8 Department of Biological Chemistry & Molecular Pharmacology, Blavatnik Institute, Harvard Medical School, Boston, MA USA; 9 CAAD Center for Translational Research on Autoimmune and Allergic Disease, University of Piemonte Orientale, Novara, Italy

**Keywords:** Herpes virus, Post-translational modifications

## Abstract

Citrullination is the conversion of arginine-to-citrulline by protein arginine deiminases (PADs), whose dysregulation is implicated in the pathogenesis of various types of cancers and autoimmune diseases. Consistent with the ability of human cytomegalovirus (HCMV) to induce post-translational modifications of cellular proteins to gain a survival advantage, we show that HCMV infection of primary human fibroblasts triggers PAD-mediated citrullination of several host proteins, and that this activity promotes viral fitness. Citrullinome analysis reveals significant changes in deimination levels of both cellular and viral proteins, with interferon (IFN)-inducible protein IFIT1 being among the most heavily deiminated one. As genetic depletion of IFIT1 strongly enhances HCMV growth, and in vitro IFIT1 citrullination impairs its ability to bind to 5’-ppp-RNA, we propose that viral-induced IFIT1 citrullination is a mechanism of HCMV evasion from host antiviral resistance. Overall, our findings point to a crucial role of citrullination in subverting cellular responses to viral infection.

## Abstract (web summary)

Citrullination is a posttranslational modification of arginines. Here, the authors show that HCMV infection increases citrullination of host and virus proteins to promote infection and that citrullinated interferon-inducible protein IFIT1 is impaired in RNA binding, as a potential mechanism of evasion.

## Introduction

Human cytomegalovirus (HCMV) is a β-herpesvirus infecting 40–90% of the adult human population. Even though HCMV infection is frequently harmless in healthy patients, it can lead to serious health consequences in individuals with a deficient immune system, such as transplant recipients and AIDS patients^1^. In addition, congenital HCMV infection is the most common cause of fetal and neonatal malformations in developed countries^2^. More recently, HCMV has been linked to autoimmune diseases and degenerative disorders, such as atherosclerosis, vascular disease, and immune aging as well as to certain types of tumors^3–8^.

One of the strategies devised by HCMV to favor its replication consists in modifying host cellular proteins at the post-translational level, thereby altering their localization, interaction, activation, and/or turnover^9^.

A post-translational modification (PTM) that is increasingly recognized to play an essential role in immune-related diseases is citrullination, also called deimination, a process where the guanidinium group of arginine is hydrolyzed to form citrulline, a non-genetically encoded amino acid^10^. This PTM is catalyzed by the calcium-dependent protein arginine deiminase (PAD) family of enzymes, which in humans is composed of five isoforms (PADs 1–4 and 6), with different tissue-specific expression and substrate specificities^11^. Although aberrant citrullination has been detected in several inflammatory conditions, such as rheumatoid arthritis (RA), systemic lupus erythematosus (SLE), Alzheimer’s disease (AD), multiple sclerosis (MS), Parkinson’s disease, cancer, and atherosclerosis, suggesting that it may play a pathogenic role in inflammation-related diseases^12–19^, a direct correlation between citrullination and viral infections has only recently emerged. In particular, human rhinovirus (HRV)-induced citrullination of LL37 was found to impair the antiviral activity of this host defense protein^20^. Consistent with the role of citrullination in viral infections, other studies have shown how artificially citrullinated Epstein–Barr virus (EBV) proteins could be specifically recognized by RA sera^21–23^. Importantly, a recent in silico analysis of transcriptome datasets from lung biopsies of SARS-CoV-2-infected patients has proposed a putative role of PADs in COVID-19^24^.

In this study, we unveil a signature of HCMV infection based on PAD-mediated citrullination of multiple cellular proteins to disrupt host -defense mechanisms.

## Results

### HCMV infection induces protein citrullination

**Fig. 1:**
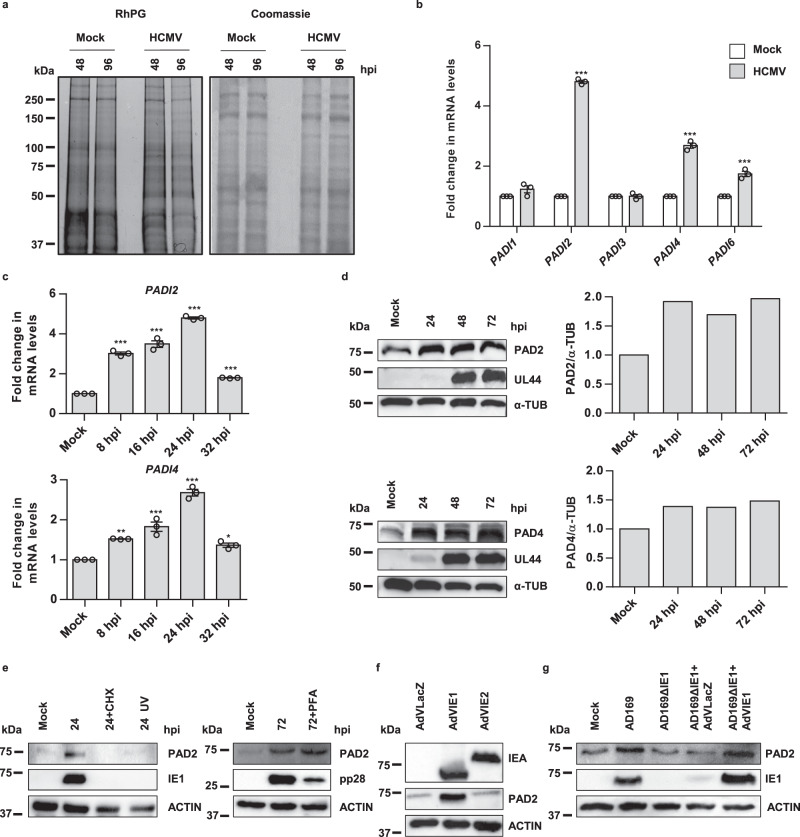
HCMV infection induces protein citrullination. **a** Protein lysates from HFFs infected with HCMV strain Merlin (HCMV) (MOI 1 PFU/cell) at 48 and 96 h post infection (hpi) or from uninfected HFFs (mock) were exposed to an Rh–PG citrulline-specific probe (left panel) and subjected to gel electrophoresis to detect citrullinated proteins. Equal loading was assessed by Coomassie blue staining (right panel). One representative gel of three independent experiments is shown. **b** mRNA expression levels of *PADI* isoforms by RT-qPCR of HCMV-infected (24 hpi) vs. uninfected (mock) HFFs were normalized to the housekeeping gene glyceraldehyde-3-phosphate dehydrogenase GAPDH and expressed as mean fold change ± SEM over mock-infected cells (*n* = 3 independent determinations; *PADI2*, *PADI4*, and *PADI6* *P* < 0.001, two-way ANOVA followed by Bonferroni’s post test). **c** *PADI2* and *PADI4* mRNA levels in HCMV-infected HFFs at the indicated time points (hpi) were normalized to GAPDH mRNA and expressed as mean fold change ± SEM over mock-infected cells (*n* = 3 indepe*n*dent determinations; *PADI2*: mock vs. 8 hpi *P* < 0.001, mock vs. 16 hpi *P* < 0.001, mock vs. 24 hpi *P* < 0.001, mock vs. 32 hpi *P* < 0.001; *PADI4*: mock vs. 8 hpi *P* = 0.00375, mock vs. 16 hpi *P* < 0.001, mock vs. 24 hpi *P* < 0.001, mock vs. 32 hpi *P* = 0.04, one-way ANOVA followed by Bonferroni’s post tests). **d** Western blot analysis of protein lysates from uninfected (mock) or infected HFFs using antibodies against PAD2, PAD4, UL44, or α-tubulin (α-TUB). One representative blot and densitometric analysis shown of three independent experiments. Values are expressed as fold change in PAD2 and PAD4 expression normalized to α-tubulin. **e** Western blot analysis of protein lysates from uninfected (mock) or infected HFFs with HCMV wild-type (HCMV), or UV-inactivated HCMV (UV) (left panel), treated with 150 μg/ml CHX (left panel) at 24 hpi, or with 250 µM PFA (right panel) at 72 hpi or left untreated. Analysis was performed using antibodies against PAD2, IEA (recognizing IE1-72- and IE2-86 kDa), pp28, or ACTIN. One representative blot of three independent experiments is shown. **f** Western blot analysis of protein lysates from uninfected (mock), infected HFFs for 48 h with AdVIE1, AdVIE2, or AdVLacZ (MOI = 10) using antibodies against IEA (recognizing IE1-72- and IE2-86 kDa), PAD2, or ACTIN. One representative blot of three independent experiments is shown. **g** Western blot analysis of protein lysates from HFFs infected with wild-type AD169 HCMV (AD169) or AD169ΔIE1 (MOI 1 PFU/cell), the latter complemented with AdVLacZ or AdVIE1 (MOI 10 PFU/cell), at 24 hpi or from uninfected HFFs (mock) using antibodies against IEA (recognizing IE1-72- and IE2-86 kDa), PAD2, or ACTIN. One representative blot of three independent experiments is shown. Data are shown as the mean ± SEM, *P < 0.05, **P < 0.01, ***P < 0.001.

To begin to characterize protein citrullination during HCMV infection, we performed immunoblot analysis of protein lysates obtained from human foreskin fibroblasts (HFFs) infected with the HCMV strain Merlin at different time points, using the citrulline-specific probe rhodamine–phenylglyoxal (Rh–PG)^25^. We observed an increase in total protein citrullination in lysates from HCMV-infected HFFs at 48 h and, albeit to a lesser extent, at 96 h post infection (hpi) compared to uninfected control cells (mock) (Fig. 1a).

We next sought to determine whether PADs played a role in enhanced citrullination by HCMV. RT-qPCR analysis revealed that *PADI2*, *4*, and *6* genes were all expressed at significantly higher levels in HCMV-infected HFFs at 24 hpi compared to mock-infected controls (i.e., ~4.8-, ~2.7, and ~1.7-fold, respectively) (Fig. 1b). In particular, both *PADI2* and *4* genes were upregulated at 8 hpi, reaching a peak 24 hpi and decreasing afterward (Fig. 1c). In contrast to their mRNA expression levels, PAD2 and 4 protein levels were already increased at 24 hpi and remained elevated for up to 72 hpi (Fig. 1d), suggesting that transcription and translation of PADs are differently regulated upon HCMV infection. By contrast, the other PAD isoforms were expressed at very low (PAD3) or undetectable levels (PAD1 and 6) and did not vary following HCMV infection (Fig. 1b and Supplementary Fig. 1a), indicating that PAD2 and 4 are the only PAD enzymes involved in HCMV-induced citrullination.

### PAD protein levels are induced by HCMV immediate early 1 (IE1) protein

To gain more insight into the mechanisms responsible for *PADI2* and *4* transcriptional upregulation, we assessed the promoter activity of both *PADI2* and *4* genes taking advantage of a dual-luciferase assay system. To this end, HFFs were transiently transfected with luciferase reporter plasmids carrying the wild-type promoter region of either *PADI2* or *PADI4*. Twenty-four hours after electroporation, cells were infected with wild-type or UV-inactivated HCMV. UV-inactivation of HCMV was confirmed by assessing its ability to produce viral immediate early antigen (IEA) compared to the intact virus (Supplementary Fig. 1b). As shown in Supplementary Fig. 1c, HCMV infection led to a robust induction of the luciferase activity driven by either the *PADI2* or *PADI4* promoter (~7- and 3.5-fold, respectively), whereas UV-HCMV infection failed to induce a similar response, indicating that the synthesis of one or more viral proteins during the initial stage of infection is critical for transcriptional activation of *PADI2* and *4* genes.

We next proceeded to determine which viral gene product was responsible for the upregulation of PAD2, the most potently induced PAD member following HCMV infection (Fig. 1b, c). Treatment of HCMV-infected HFFs with the protein synthesis inhibitor cycloheximide (CHX) completely shut down HCMV-induced PAD2 protein expression at 24 hpi, attesting that de novo expression of viral proteins is required to upregulate PAD2 (Fig. 1e). Fittingly, UV-inactivated HCMV failed to induce PAD2 (Fig. 1e). In contrast, treatment with the viral DNA synthesis inhibitor phosphonoformic acid (PFA) did not seem to affect PAD2 upregulation (Fig. 1e), indicating that true late viral proteins are not involved in PAD regulation.

These results, together with the observation that the early kinetics of PAD induction (Fig. 1c) paralleled that of viral IE1 or IE2 gene expression, raised the hypothesis that IE gene products may play a functional role in PAD induction. Indeed, adenoviral-mediated overexpression of IE1 (AdVIE1) but not IE2 (AdVIE2) led to a substantial upregulation of both PAD2 protein expression (Fig. 1f), indicating that IE1 is at least one of the HCMV products regulating PAD gene expression. Consistently, HFFs infected with a recombinant HCMV strain lacking the IE1 protein (AD169ΔIE1) displayed much lower levels of PAD2 protein expression at 48 hpi compared to cells transduced with the wild-type control (AD169), an effect that was reversed by AdVIE1 co-infection (Fig. 1g)^26^. In line with previous results, induction of citrullination by the AD169 strain was also confirmed using the citrulline-specific probe Rh–PG (Supplementary Fig. 1d).

### PAD inhibition blocks HCMV replication

**Fig. 2:**
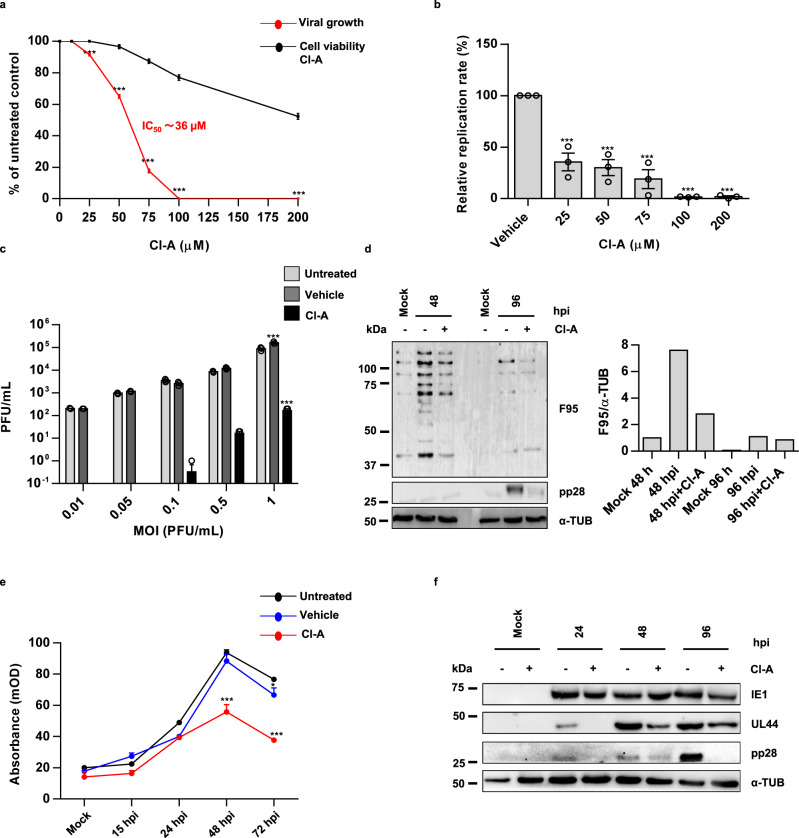
Cl-A blocks HCMV replication. **a** HFFs were infected with HCMV (MOI 0.1 PFU/cell) and then treated with increasing concentrations of Cl-A, which were given 1 h prior to virus adsorption and kept throughout the whole experiment. At 144 hpi, viral plaques were microscopically counted and expressed as a percentage relative to untreated controls. The number of plaques is plotted as a function of Cl-A concentration. The concentrations resulting in 50% plaque formation (IC_50_) reduction are represented by the red line. The number of viable cells was determined for each Cl-A concentration using the MTT method (black line). Values are expressed as means ± SEM (error bars) of three independent experiments (untreated vs. 25 µM Cl-A *P* < 0.001, untreated vs. 50 µM Cl-A *P* < 0.001, untreated vs. 75 µM Cl-A *P* < 0.001, untreated vs. 100 µM Cl-A *P* < 0.001, untreated vs. 200 µM Cl-A *P* < 0.001, one-way ANOVA followed by Bonferroni’s post test). **b** To determine the number of viral DNA genomes in HCMV-infected HFFs, viral DNA was isolated at 144 hpi and analyzed by qPCR using primers amplifying a segment of the IE1 gene. GAPDH was used to normalize HCMV genome counts. Values are expressed as mean ± SEM of three independent experiments (vehicle vs. 25 µM Cl-A *P* < 0.001, vehicle vs. 50 µM Cl-A *P* < 0.001, vehicle vs. 75 µM Cl-A *P* < 0.001, vehicle vs. 100 µM Cl-A *P* < 0.001, vehicle vs. 200 µM Cl-A *P* < 0.001, one-way ANOVA followed by Bonferroni’s post test). **c** HFFs were infected with HCMV at increasing MOIs (0.01–1 PFU/cell) and then treated with 100 µM Cl-A or vehicle. Viral supernatants were collected at 144 hpi and analyzed by standard plaque assay. Values are expressed as mean ± SEM of three independent experiments (MOI 1: untreated vs. vehicle *P* < 0.001, untreated vs. Cl-A *P* < 0.001, two-way ANOVA followed by Bonferroni’s post test). **d** Protein lysates from uninfected (mock) or infected HFFs (48 and 96 hpi) with (+) or without (−) 100 µM Cl-A were subjected to immunoblotting using the anti-peptidylcitrulline F95 antibody to detect citrullinated proteins, anti-pp28 to assess HCMV infection, or anti-α-tubulin (α-TUB) to show equal loading. The densitometric analysis shown is representative of three independent experiments. Densitometry values of F95 are normalized to those of α-tubulin. **e** PAD enzymatic activity assay. Histone H3 was immobilized on a 96-well microtiter plate and incubated with protein lysates from HCMV-infected HFFs or uninfected (mock) at the indicated time points, in the presence (red line) or absence (untreated or vehicle alone, black and blue line, respectively) of Cl-A. The conversion of peptidylarginine to peptidylcitrulline was detected with an anti-H3 citrulline antibody. Detection of the bound antibodies was performed by ELISA. Values are expressed as means ± SEM (error bars) of three independent experiments (48 hpi: untreated vs. Cl-amidine P < 0.001, 72 hpi: untreated vs. Cl-A *P* < 0.001, 72 hpi: untreated *vs.* vehicle *P* < 0.05, two-way ANOVA followed by Bonferroni’s post tests). **f** Protein lysates from uninfected (mock) or infected HFFs (24, 48, or 72 hpi) at an MOI of 1 PFU/cell treated with or without Cl-A ( + ) or vehicle (−) were analyzed by immunoblotting for viral expression (IE1-72 kDa, UL44, and pp28) and normalized to α-tubulin (α-TUB). One representative blot of three independent experiments is shown. Data are shown as the mean ± SEM, *P < 0.05, **P < 0.01, ***P < 0.001.

Next, we asked whether citrullination induced by HCMV infection would affect viral replication. To answer this question, we assessed viral plaque formation in HCMV-infected HFFs (MOI 0.1) treated for 1 h prior to infection with increasing concentrations of Cl-amidine (Cl-A) (25–200 µM), a cell-permeable pan-PAD inhibitor^27^. After 7 days of continuous exposure to Cl-A, we observed a dose-dependent downregulation in the number of viral particles in HCMV-infected HFFs, with a complete suppression at 100 µM. Assessment of cell cytotoxicity by means of the 3-(4,5-dimethylthiazol-2-yl)-2,5-diphenyltetrazolium bromide (MTT) assay in HFFs treated under the same conditions ruled out the possibility that the antiviral activity was related to the cytotoxic effects of the drug (Fig.2a). The half-maximal inhibitory concentration (IC_50_) of Cl-A was found to be 36 µM (Fig. 2a). To further corroborate these data, we also measured viral DNA synthesis by quantitative real-time PCR (qPCR) in similarly treated cells. Remarkably, at the low concentration of 25 µM, Cl-A treatment reduced the rate of HCMV DNA replication by approximately threefold compared to untreated cells (Fig. 2b). In line with the plaque-forming activity data, treatment with 100 µM Cl-A completely shut down HCMV DNA replication (Fig. 2b). Next, HFFs were treated for 1 h prior to HCMV infection with 100 µM Cl-A, infected with increasing MOI (0.01–1) of HCMV, and incubated for an additional 144 h in the presence of the inhibitor. At 144 hpi, the number of plaque-forming units (PFUs) was completely suppressed at MOIs ranging from 0.01 to 0.1 (Fig. 2c). Interestingly, Cl-A maintained its antiviral effect even at higher HCMV MOIs (0.5–1). Consistently, this remarkable reduction in the number of viral particles was observed also in HCMV-infected HFFs treated with BB-Cl-amidine (BB-Cl-A)^14^, a second-generation pan-PAD inhibitor, at nontoxic concentrations much lower than Cl-A of 1 and 0.5 µM, with a complete suppression at 1 µM (Supplementary Fig. 2a).

To determine whether the antiviral activity of the compound was limited to HCMV or could be extended to other viruses, we assessed the effect of increasing Cl-A concentrations on the replication of: (i) herpes simplex virus 1 (HSV-1) and 2 (HSV-2), two members of the Herpesviridae family; (ii) a clinical isolate of adenovirus as a prototype of a non-enveloped DNA virus; and (iii) the RNA virus human immunodeficiency virus 1 strain IIIb (HIV-1_IIIb_). Interestingly, only HSV-1 and -2 displayed impaired viral growth in Cl-A-treated cells (IC_50_ ∼66 and 21 μM) (Supplementary Fig. 2b), whereas the replication rates of adenovirus and HIV-1_IIIb_ were only marginally affected by the PAD inhibitor (Supplementary Fig. 2c). Of note, the slight reduction in adenovirus replication observed in HFFs cells treated with higher doses of Cl-A was probably due to the cytotoxic effect of the compound, as judged by the MTT assay.

To further demonstrate that Cl-A reduces HCMV-driven citrullination in HFFs, we assessed total protein citrullination levels using an anti-peptidylcitrulline antibody (clone F95). In line with the results obtained with the Rh–PG probe (Fig. 1a), protein citrullination peaked at 48 hpi and decreased at 96 hpi (Fig. 2d). As expected, in the presence of Cl-A, protein citrullination was efficiently suppressed at either time points (Fig. 2d). Next, PAD enzymatic activity was measured by means of an in vitro antibody-based assay using histone 3 as a substrate (Fig. 2e). The reliability of the assay was first assessed using increasing amounts of human recombinant PAD2 in the presence or absence of Cl-A (Supplementary Fig. 1e). Consistent with the kinetics of PAD2 and PAD4 protein expression and with the citrullination profile, PAD catalytic activity was enhanced at 24 hpi, peaked at 48 hpi, and decreased at 72 hpi. Importantly, HCMV-induced PAD activity was significantly inhibited by Cl-A (100 µM) at 48 and 72 hpi (Fig. 2e). Finally, to assess the extent of HCMV replication, total protein extracts from HCMV-infected HFFs treated with or without Cl-A at various time points post infection were subjected to immunoblotting using antibodies against the corresponding IEA, early (UL44), and late (pp28) proteins. While Cl-A treatment only marginally affected IEA expression, it inhibited the expression of viral early and late genes (Fig. 2f), indicating that PAD enzymes support the HCMV productive cycle by fostering the expression of early and late genes.

**Fig. 3:**
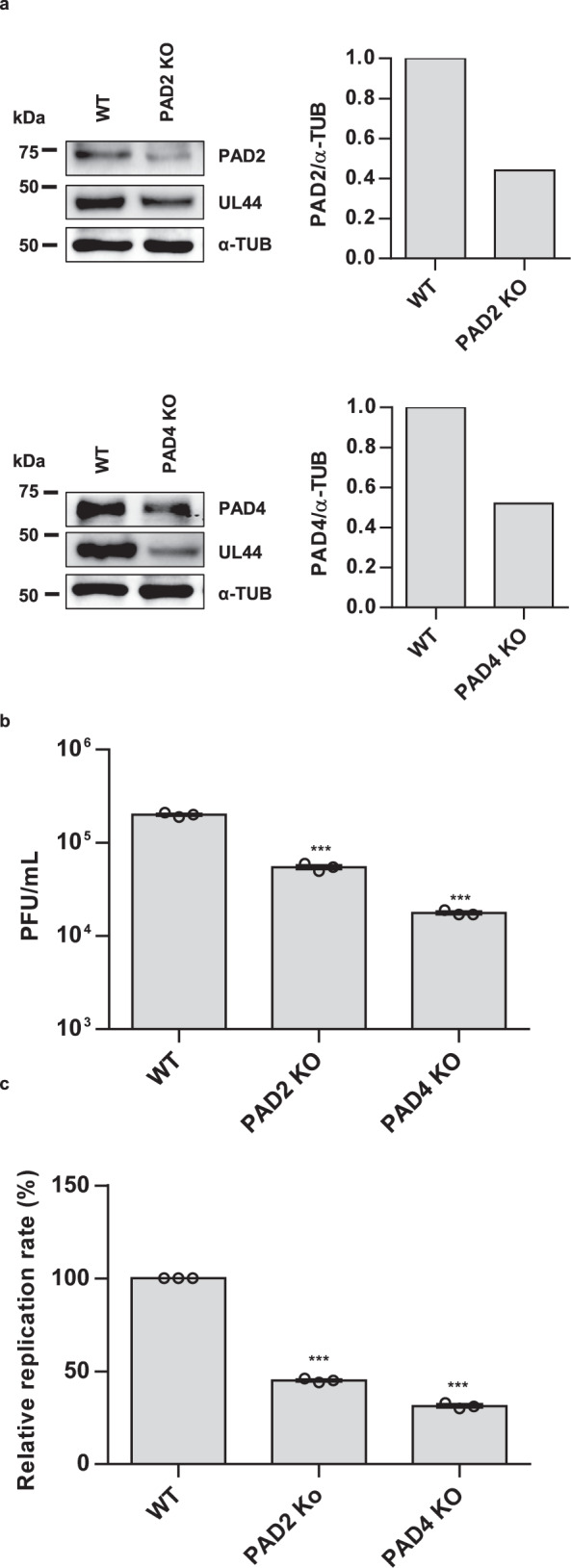
Effect of PAD2 and PAD4 gene knockout on HCMV replication. **a** Knockout (KO) gene variants in HFFs for PAD2 (PAD2 KO) and PAD4 (PAD4 KO) were generated using CRISPR/Cas9 technology. The efficiency of PAD2 and PAD4 protein depletion at 48 hpi was assessed by immunoblotting using antibodies against PAD2, PAD4, or α-tubulin (α-TUB), for equal loading. An anti-UL44 antibody was used to verify HCMV infection. The western blot and relative densitometric analysis are representative of three independent experiments. Values are expressed as fold change in PAD2 and 4 expression normalized to α-tubulin (α-TUB). **b** HFFs KO cells were infected with HCMV at an MOI of 0.1 PFU/cell. Viral supernatants were collected at the indicated time points and analyzed by standard plaque assay. Values are expressed as means ± SEM of three independent experiments (WT vs. PAD2 KO *P* < 0.001, WT vs. PAD4 KO *P* < 0.001, one-way ANOVA followed by Bonferroni’s post test). **c** To determine the number of viral DNA genomes in HCMV-infected HFFs KO cells (MOI 0.1), viral DNA was isolated at 144 hpi and analyzed by qPCR with primers amplifying a segment of the IE1 gene. GAPDH was used to normalize HCMV genome counts. Values are expressed as mean ± SEM of three independent experiments (WT vs. PAD2 KO *P* < 0.001, WT vs. PAD4 KO *P* < 0.001, one-way ANOVA followed by Bonferroni’s post test). Data are shown as the mean ± SEM, *P < 0.05, **P < 0.01, ***P < 0.001.

To confirm that the antiviral activity of Cl-A was due to PAD inhibition and not to an off-target effect of the compound, we generated PAD knockout (KO) HFFs using clustered regularly interspaced short palindromic repeat (CRISPR)/Cas9 technology. Primary cell lines carrying mutations in genes encoding PAD1 (PAD1 KO), PAD2 (PAD2 KO), PAD3 (PAD3 KO), PAD6 (PAD6 KO), or PAD4 (PAD4 KO) were generated using five different guide RNAs (gRNAs). Tracking of indels by decomposition (TIDE) analysis showed an overall knockdown efficiency ranging from 35 to 45% for each of the PAD KO cell lines (Supplementary Fig. 3). Consistently, immunoblot analysis revealed a reduction in PAD2, 4, and 3 protein expression signals between 50 and 60% (Fig. 3a and Supplementary Fig. 4a). With regard to PAD1 and 6, we could only rely on the aforementioned TIDE analysis (Supplementary Fig. 3) as the expression levels of these two PAD isoforms were barely detectable in HFFs, even after HCMV infection (Supplementary Fig. 1a). Although we failed to achieve total suppression of PAD gene expression, the overall knockdown efficiency proved to be sufficient enough to allow us to perform subsequent functional experiments.

To assess whether PADs are required for HCMV replication, we performed a standard plaque assay and qPCR as described above. As shown in Fig. 3b, c, PAD2 and PAD4 KO cells, respectively, displayed 0.5 and 1 logs of viral load reduction, and ~60 and 70% decrease in viral replication, at an MOI of 0.1. In line with these results, a consistent decrease in UL44 protein expression levels was also detected in infected PAD2, and PAD4 KO cell lines at 48 hpi (Fig. 3a). As expected, depletion of PAD1 (PAD1 KO), 3 (PAD3 KO), or 6 (PAD6 KO) in HFFs did not affect the HCMV replication rates compared to wild-type control cells (Supplementary Fig. 4b, c). Thus, PAD2 and 4 but not PAD1, 3, and 6 sustain the viral cell cycle of HCMV. These results were further confirmed in HFFs transfected with siRNA against PAD2 and PAD4, where a protein knockdown efficiency of ~100 and 70%, respectively (Supplementary Fig. 4d), resulted in an even higher viral reduction of 1 and 2 logs, respectively (Supplementary Fig. 4e). In addition, while siRNA control-transfected HFFs treated with 100 µM Cl-A showed a 1-log reduction in the viral load, no significant differences were observed in PAD2- or PAD4-depleted cells (Supplementary Fig. 4e), indicating no predominant role of one isoform over the other in our working model.

### IFN-inducible proteins are the main targets of PAD-mediated citrullination

**Fig. 4:**
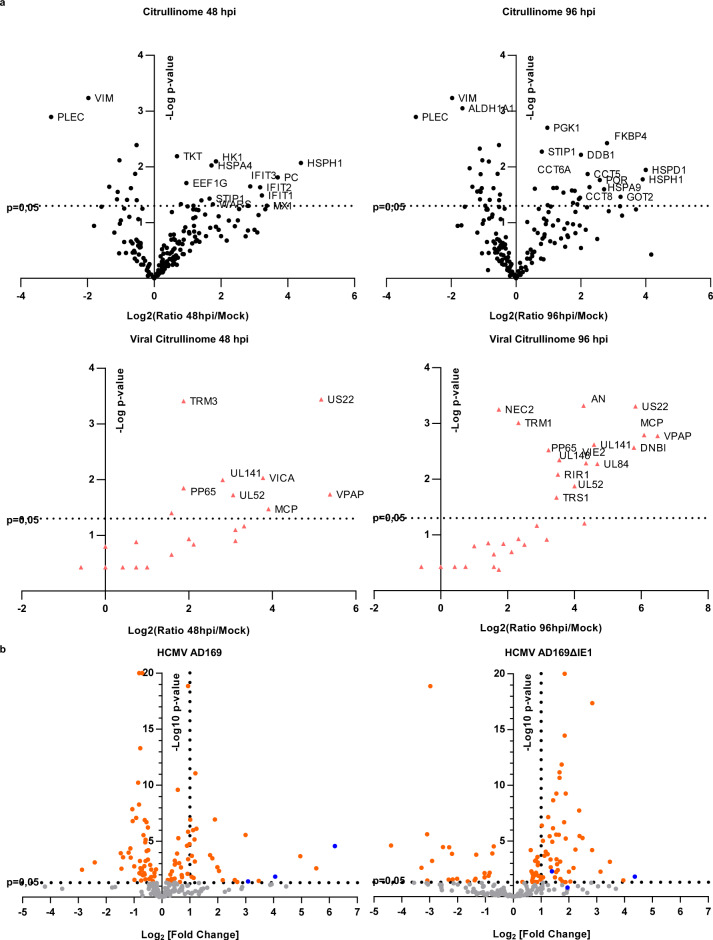
Characterization of the citrullinated proteome (citrullinome) of HCMV-infected cells. **a** Volcano plot depicting the host (upper panel—circle) and viral (lower panel—triangle) citrullinated proteins of infected cells vs. mock-infected cells at 48 hpi (left panel) and 96 hpi (right panel). Cell lysates from uninfected (mock) or HCMV-infected HFFs (MOI 1) were exposed to a biotin-PG to isolate citrullinated proteins on streptavidin agarose. Bound proteins were then subjected to on-bead tryptic digestion and analyzed by LC-MS/MS—in the graph, every identified citrullinated protein corresponds to a dot. The *x* axis represents the ratio of citrullination between mock and infected cells at the indicated time points, while the *y* axis indicates the statistical significance. Both variables were plotted on a logarithmic scale (*n* = 3). **b** Volcano plot depicting the host citrullinated proteins in HCMV AD169- (left panel) and AD169ΔIE1- (right panel) vs. mock-infected HFFs at 48 hpi. Cell lysates from uninfected (mock) or HCMV-infected (MOI 1) HFFs were exposed to a biotin-PG to isolate citrullinated proteins on streptavidin agarose. Bound proteins were then subjected to on-bead tryptic digestion and analyzed by LC-MS/MS—in the graph, every identified citrullinated protein corresponds to a dot. The *x* axis represents the ratio of citrullination between mock and infected cells at the indicated time points, while the *y* axis indicates the statistical significance. Both variables were plotted on a logarithmic scale (*n* = 3).

To identify which proteins (i.e., cellular and/or viral) were deiminated during HCMV infection, we performed citrullinome analysis on HCMV-infected cells harvested at 48 and 96 hpi. Consistent with our earlier findings, we observed a massive increase in overall protein citrullination in HCMV-infected cells at both 48 and 96 hpi compared to uninfected control cells, even though citrullination levels were slightly diminished at 96 hpi compared to 48 hpi (Fig. 4a and Supplementary Data 1, 2, and 3), confirming our previous results (Fig. 1a). In addition to detecting numerous citrullinated viral proteins—35 at 48 hpi and 40 proteins at 96 hpi—we noticed an even higher number of citrullinated host cell proteins—177 at 48 hpi and 122 at 96 hpi. Using PANTHER software, we were able to identify a wide range of citrullinated host proteins falling into various functional classes, among which cytoskeletal proteins, chaperones, oxidoreductase, hydrolase, and nucleic acid binding proteins were more frequently found at both time points of infection (Supplementary Fig. 5a). Of particular interest was the significant citrullination at 48 hpi of several members of the interferon (IFN)-induced protein with tetratricopeptide repeat (IFIT) family, such as IFIT1, IFIT2 and IFIT3, and of the IFN-inducible myxovirus resistance 1 (Mx1) gene product (Fig. 4a, left panel)—at 96 hpi, citrullination of these proteins was no longer detectable (Fig. 4a, right panel).

To validate these findings, total proteins from mock or infected HFFs at 48 hpi were immunoprecipitated with the anti-citrulline F95 antibody and subjected to immunoblotting using antibodies against Mx1, IFIT1, IEA, UL44, and pp65. As shown in Supplementary Fig. 5b, all five proteins were robustly deiminated following infection with HCMV.

To corroborate our finding that IE1 is crucial for PAD induction and subsequent citrullination, we performed an additional citrullinome analysis on uninfected HFFs, and wild-type AD169, or mutant AD169ΔIE1-infected HFFs at 48 hpi (Fig. 4b and Supplementary Data 4 and 5). Consistent with previous results, we detected a significant overall protein citrullination in AD169-infected cells at 48 hpi, with IFIT1, IFIT2, and IFIT3, and Mx1 being among the most highly deiminated cellular proteins compared to uninfected control cells (Fig. 4b, left panel). In contrast, upon infection with AD169ΔIE1, we observed a 6.3-, 3.5-, and 2.2-fold decrease in IFIT1, Mx1, and IFIT3 enrichment, respectively (Fig. 4b, right panel), which further supports the crucial role played by the IE1 protein in PAD-mediated citrullination during HCMV infection.

### The IFN-inducible proteins IFIT1 and Mx1 exert antiviral activity against HCMV

IFITs are a family of antiviral RNA-binding proteins highly expressed during antiviral immune responses. In this regard, IFIT family members, known primarily for their antiviral activity against RNA viruses, have only recently been implicated in the innate immune response against DNA viruses^28^. Specifically, Li and Swaminathan^29^ have shown that human IFIT1, IFIT2, and IFIT3 proteins can suppress lytic replication of the Kaposi’s sarcoma-associated herpesvirus (KSHV). Furthermore, Zhang et al. demonstrated that IFIT1 overexpression significantly impairs HCMV replication in astrocytes, whereas IFIT1 knockdown sustains the viral cycle of HCMV^30^.

The other IFN-inducible target of HCMV-induced deimination, Mx1, is a member of the dynamin-like large GTPase family involved in protection against negative-stranded RNA virus infection and several DNA viruses^31^. Interestingly, MX2 has been recently shown to display antiviral activity against herpesviruses^32,33^.

**Fig. 5:**
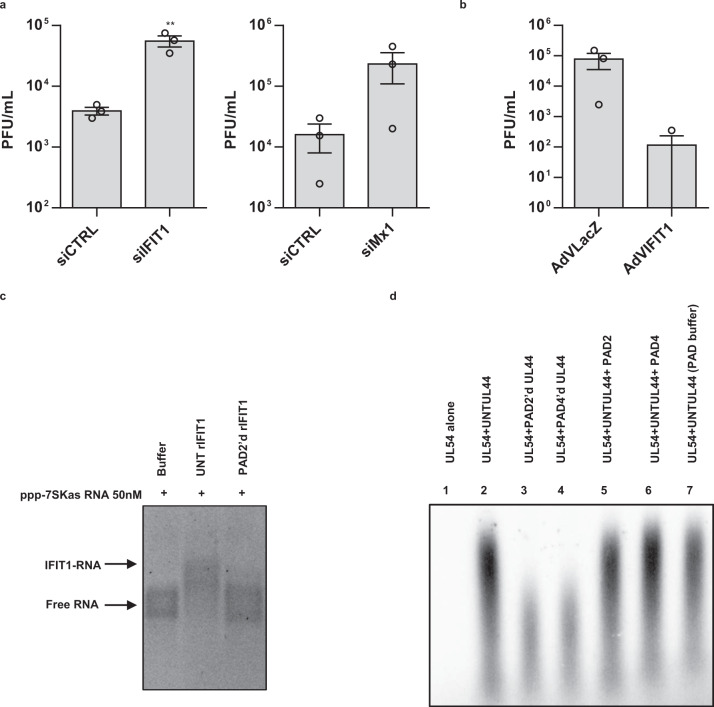
The antiviral role of IFIT1 and Mx1 against HCMV. **a** HFFs were silenced for IFIT1 and Mx1 using specific siRNAs (siIFIT1, siMx1, respectively). As negative control cells were also similarly transfected with scrambled siRNA (siCTRL). At 24 hpt, cells were infected with HCMV at an MOI of 0.1 PFU/cell. Viral supernatants were collected at 144 hpi and analyzed by standard plaque assay. Values are expressed as means ± SEM of three independent experiments (siCTRL vs. siIFIT1 *P* = 0.0111, unpaired two-tailed *t* test; siCTRL vs. siMx1 *P* > 0.05, unpaired two-tailed *t* test). **b** HFFs were transduced with AdVIFIT1 or AdVLacZ at an MOI of 10 PFU/cell. Subsequently, cells were infected with HCMV at an MOI of 1. The extent of virus replication was measured at 144 hpi. Results are expressed as means ± SEM of three independent experiments (AdVLacZ vs. AdVIFIT1 *P* > 0.05, unpaired two-tailed *t* test). **c** Gel electrophoretic mobility shift assay of IFIT1 binding to PPP-7SKas RNA. 50 nM of in vitro transcribed 7SK 5’-ppp-RNA was incubated with only buffer (buffer), 5 µM of recombinant IFIT1, either untreated (UNT rIFIT1) or treated with PAD2 (PAD2’d rIFIT1) and loaded on a tris-glycine agarose gel. Data are representative of three experiments. **d** Long-chain DNA synthesis directed by UL54 in the presence or absence of purified UL44ΔC290 was assayed by measuring the incorporation of labeled [_32_P]TTP with a poly(dA)-oligo(dT) primer template. DNA products were resolved on an alkaline agarose gel that was exposed to film and, for quantification, to a phosphorescence screen followed by scanning with a Typhoon scanner. The image shows products directed by UL54 alone, lane 1), or by UL54 in the presence of untreated (UNT) UL44ΔC290 (lane 2), in the presence of UL44ΔC290 treated with PAD2 or PAD4 (PAD2’d, PAD4’d; lanes 3 and 4, respectively), in the presence of UNT UL44ΔC290 but with 270 pM PAD2 (lane 5) or 370 pM PAD4 (lane 6) added to the DNA synthesis reaction, or in the presence of UL44 incubated in PAD reaction buffer, but without PADs (mock-PAD, lane 7). One representative gel of three independent experiments is shown. Data are shown as the mean ± SEM, *P < 0.05, **P < 0.01, ***P < 0.001.

To gain further insight into the role of these genes during HCMV infection, we measured virus production in HCMV-infected HFFs after siRNA-mediated depletion of Mx1 or IFIT1—the latter having been shown to exert antiviral activity against HCMV only in fetal astrocytes upon lentiviral overexpression^30^. Following transfection with specific siRNAs against IFIT1 or Mx1 (siIFIT1, siMx1) and subsequent infection with HCMV for 48 h, we achieved a ~60% reduction in IFIT1 protein expression and near to complete silencing of Mx1 protein (Supplementary Fig. 6a). Importantly, in both cases, gene silencing resulted in 1-log higher levels of virus production compared to siCTRL-transfected cells (Fig. 5a).

Given the emerging evidence supporting an antiviral role of IFIT1 against HCMV infection, we asked whether ectopic expression of IFIT1 would curb viral spread in our system. To test this hypothesis, HFFs were transduced with AdVIFIT1 or LacZ as a control at an MOI of 10 and, 24 h post transduction (hpt), infected with HCMV at an MOI of 1 for 144 h. As expected, overexpression of IFIT1 (Supplementary Fig. 6b) reduced viral titer by >2-log compared to AdVLacZ control (Fig. 5b), indicating that IFIT1 strongly impairs HCMV infection in HFFs.

### In vitro citrullination of IFIT1 inhibits its binding to triphosphorylated RNA

In order to elucidate the effect of citrullination on IFIT1, we exploited its well-established ability to preferentially bind viral RNA harboring a triphosphate group at its C-terminus (5’-ppp-RNA)^34^, which has been shown to be detrimental for the growth of some RNA viruses containing 5’-ppp-RNA. Interestingly, the arginine at position 187 in a highly charged carboxy-terminal groove appears necessary for IFIT1 binding^35^. In this context, we first attempted to determine whether this arginine 187 could be citrullinated by PAD2 after assessment of its in vitro citrullination using the Rh–PG (Supplementary Fig. 6c). LC-MS/MS peptide analysis of recombinant IFIT1 mock or treated with PAD2 for 3 h identified several arginine positions citrullinated in vitro by PAD2, including arginine 187 (Supplementary Data 6). We then conducted a gel shift assay to assess the ability of IFIT1 to bind to 5’-ppp-RNA according to its citrullination state. In vitro transcribed 5’-ppp-RNA was incubated with recombinant IFIT1, either mock or treated with PAD2, and then loaded on an agarose gel. As expected, mock IFIT1 induced a delay in 5’-ppp-RNA migration in comparison with control RNA, indicating the formation of an IFIT1-RNA complex. This effect was reduced when IFIT1 was previously treated with PAD2 for 3 h (Fig. 5c).

### In vitro citrullination of UL44 affects long-chain DNA synthesis

To determine whether citrullination could also affect viral protein activity, we measured the ability of native vs. citrullinated forms of VPAP, also known as ICP36 or UL44, to cooperate with the viral DNA polymerase UL54 in promoting long-chain DNA synthesis^36,37^, using poly (dA)-oligo (dT)_12-18_ as primer template (Fig. 5d, and Supplementary Fig. 6d). The choice of VPAP was due to its high deimination rate observed at both 48 and 96 hpi compared to mock control (Fig. 4a, and Supplementary Data 1 and 3). To perform this assay, we employed a truncated form of UL44 (UL44ΔC290) retaining all known biochemical activities of full-length UL44 for DNA polymerase stimulation of full-length UL44^38^. As expected, in the presence of UL54 alone we could not detect long-chain DNA, whereas long-chain DNA synthesis was readily detected when both UL54 and UL44ΔC290 were added to the reaction (Fig. 5d and Supplementary 6d). Notably, PAD2- or PAD4-mediated citrullination of UL44ΔC290 reduced the synthesis of long-chain DNA substantially (Fig. 5d and Supplementary Fig. 6d). This inhibition was not due to carryover of PAD2 or PAD4 because addition of PAD2 and 4 to the reaction did not inhibit DNA synthesis (Fig. 5d and Supplementary 6d). Finally, in vitro citrullination of UL44ΔC290 was confirmed by reduced mobility on SDS-PAGE compared to untreated or mock-treated controls (Supplementary Fig. 6e), as shown previously for other citrullinated proteins^39^.

## Discussion

Citrullination is an irreversible PTM catalyzed by PADs enzymes. This modification results in the conversion of an arginine side chain into citrulline, with loss of one positive charge and consequent reduction in protein net charge. This PTM can in turn increase protein hydrophobicity and alter intra- and intermolecular interactions, affecting protein conformation, stability, and binding of a wide range of proteins involved in numerous physiological processes, such as apoptosis, differentiation, and gene regulation^10^. Eventually, these structural changes can lead to the gain or loss of protein functions^40^. In this study, we have uncovered the role of citrullination in promoting viral persistence through the deimination of cellular proteins endowed with antiviral activity. Among these, we have focused our attention on IFIT1 and Mx1, two IFN-stimulated genes (ISGs) with a well-established inhibitory activity against various DNA and RNA viruses^28–33^. We show that silencing of either gene results in augmented virus production, whereas ectopic expression of IFIT1 inhibits viral growth, in good agreement with previous results obtained using KSHV-infected cells^29^. Finally, we report that PAD-mediated in vitro deimination of IFIT1 severely affects its ability to bind to 5’-triphosphorylated RNA, thereby impairing the antiviral activity of this protein.

Taken together, our findings suggest that HCMV can trigger PAD-mediated deimination of arginine residues of cellular proteins in order to enhance viral growth, a possibility supported by previous work showing that the arginine at position 187 of the IFIT1 protein is necessary for its binding to 5’-triphosphorylated RNA^34^. This residue is located within a highly charged carboxy-terminal groove that allows recognition and sequestration of viral nucleic acids. Thus, in future studies it will be crucial to characterize the yet unknown mechanisms of IFIT1- and Mx1-mediated inhibition of HCMV growth and to ascertain whether citrullination can hijack these defense pathways in vivo.

Noteworthy, we demonstrate that HCMV IE1 protein is a pivotal factor for PAD induction and subsequent protein citrullination. In this regard, our citrullinome analysis reveals that a variety of cellular proteins, such as IFITs or Mx1, are significantly less citrullinated upon infection with the AD169ΔIE1 mutant compared to the wild-type virus, implying a central role of IE1 in mediating HCMV-induced citrullination events. The residual amount of citrullinated—IFN-inducible—proteins still detectable upon infection with AD169ΔIE1 suggests that other (viral) proteins may be implicated in PAD induction or that a small number of inactive PADs may be present inside the cell and still capable of citrullinating certain targets upon HCMV-mediated activation. Our findings are consistent with a recent study by Casanova et al. showing that HRV infection upregulates PAD activity and that citrullination of the human cathelicidin LL-37 suppresses its antiviral activity against HRV infection, corroborating the role of citrullination as a viral evasion mechanism employed by certain viruses. Noteworthy, citrullination of this host defense peptide has been shown to disrupt its antimicrobial role, both against bacterial and viral infections, including HRV, and increase its susceptibility to degradation^41–46^.

Another important observation of our study is that, upon cellular entry, several viral proteins are citrullinated as well, raising the hypothesis that citrullination may concomitantly influence the activity of both viral and cellular proteins. Among viral proteins, we find that in vitro citrullination of UL44, an accessory protein that increases the DNA processivity of UL54^36,37^, reduces its ability to drive long-chain DNA synthesis. Although the physiological role of UL44 citrullination during HCMV infection cannot be inferred from the results of this study, it is tempting to speculate that HCMV may exploit UL44 citrullination to fine-tune its DNA replication during specific phases of viral infection.

Overall, our observation that HCMV growth relies, in part, on PAD-mediated citrullination suggests the attractive prospect that deimination of host—and possibly—viral proteins may be exploited for the design of antiviral agents against HCMV and, more in general, herpesviruses. An alternative pharmacological strategy—perhaps less costly and time-consuming—could rely on repurposing existing PAD inhibitors, such as Cl-A, which we have shown to have a potent inhibitory effect against HCMV-induced citrullination and viral growth.

As HCMV has been implicated in various autoimmune, inflammatory, and cardiovascular diseases as well as cancer development, all characterized by a high degree of protein citrullination, it is possible that HCMV-mediated protein citrullination also constitutes a key event in the pathogenesis of such diseases.

## Methods

### Cells and viruses

Human foreskin fibroblasts (HFFs, ATCC SCRC-1041™), African green monkey kidney cells (Vero; Sigma-Aldrich 84113001), human HEK 293 from the human kidney (embryonic) (HEK 293, Sigma-Aldrich 85120602), HEK 293T (HEK 293T, ATCC^®^ CRL-3216™) were cultured in Dulbecco’s modified Eagle’s medium (DMEM) supplemented with 10% fetal calf serum (FCS) (Sigma-Aldrich, Milan, Italy). CD4 + lymphoblastoid T (C8166, ECACC 88051601) were kept in RPMI 1640 (GIBCO, Grand Island, NY) supplemented with 10% FCS (Sigma-Aldrich, Milan, Italy).

HCMV strain Merlin, kindly provided by Gerhard Jahn and Klaus Hamprecht (University Hospital of Tübingen, Germany), and the HCMV laboratory strain AD169 (ATCC-VR538), were propagated and titrated on HFFs^47^. AD169ΔIE1 was kindly provided by Thomas Stamminger (University of Ulm, Germany). UV-inactivated Merlin was prepared using a double pulse of UV-B light (1.2 J/cm^2^).

A clinical isolate of adenovirus was propagated in HEK 293 cells, whereas clinical isolates of HSV-1 and HSV-2 were grown in Vero cells and titrated by standard plaque assay^48^. HIV-1_IIIb_ strain stock was prepared in C8166 cells, as previously described^49^.

Recombinant adenoviral vectors (AdV) encoding HCMV IE2 (AdVIE2) and *Escherichia coli* β-galactosidase (AdVLacZ) have been previously described^47,50^, while AdV-IE72 (AdVIE1) was provided by Dr. Timothy F. Kowalik (University of Massachusetts Medical School, Worcester, MA)^51^. Recombinant AdV stocks were propagated and titrated in HEK 293 by standard plaque assay^47,50,51^.

### Reagents and proteins

Recombinant human PAD2, PAD4, and Cl-A were from Cayman Chemical (Ann Arbor, USA). Cycloheximide and Foscarnet were from Sigma-Aldrich (Milan, Italy). BB-Cl-A was kindly provided by P. R. Thompson (University of Massachusetts, Medical School).

The plasmid pET30A hIFIT1-GST-His was kindly provided by A. Pichlmair (Technische Universitaet Munich, Germany). pSGIE72 IE1-encoding plasmid was used in the quantitative nucleic acid analysis. The pSicoR-CRISPR-Cas9 vector (RP-557) was kindly provided by R. J. Lebbink (UMC, Utrecht).

### In vitro antiviral assay

HFFs, HEK 293, and Vero were incubated with increasing concentrations of Cl-A (0, 25, 50, 75, 100, or 200 µM) 1 h prior to being infected with HCMV, adenovirus or HSV-1 or HSV-2 at an MOI of 0.1. Following virus adsorption (2 h at 37 °C), cultures were maintained in medium containing the corresponding Cl-A and then incubated until control cultures displayed extensive cytopathology (7 days pi for HCMV, 6 days pi for adenovirus, and 48 hpi for HSV-1 and HSV-2). Thereafter, the cells and supernatants from the antiviral assay were harvested and disrupted by sonication. The extent of virus replication was then assessed by titrating the infectivity of supernatants by standard plaque assay on HFFs for HCMV, on HEK 293 for adenovirus, and on Vero cells for HSV-1/HSV-2^47,48^.

### Cell viability assay

To determine cell viability, HFFs, HEK 293, and Vero were exposed to increasing concentrations of Cl-A. After 6 days of incubation, the number of viable cells was determined by the 3-(4,5-dimethylthiazol-2-yl)-2,5-diphenyltetrazolium bromide (MTT) method^52^. Briefly, MTT solutions from the Stock (final concentration 500 µg/ml) were added, and cells were incubated in a CO_2_ incubator in the dark for 2 h. Then, the medium was removed and the resulting formazan crystals were dissolved using 100 µl of DMSO. Finally, the resulting colored solution was transferred in a 96-well plate, and the absorbance was read at 570 nm using 630 nm as reference wavelength on a Victor X3 Multilabel Plate Reader (Perkin Elmer, Waltham, MA, USA; wallac 1420 work station software). The viability of the C8166 cells in presence of scalar concentrations of Cl-A was determined by the trypan blue exclusion technique at day 7 pi.

### HIV-1 infection and antiviral assay

HIV-1_IIIb_ (5 ng/ml of HIV-1 gag p24) was pre-incubated for 1 h at 37 °C with the increasing amount of Cl-A (0, 10, 25, 75, 100, or 200 µM) and then added to C8166 cells (0.5 × 10^6^ cells/ml) for 2 h at 37 °C. After three washes in 1X phosphate-buffered saline (PBS), cells were seeded at 5 × 10^5^ cells/ml into a fresh medium plus the same drug concentration used in the pre-incubation. The HIV-1 gag p24 amount was determined at day 7 pi in the culture supernatants with the HIV-1 p24 antigen ELISA kit (Biomerieux, Marcy-l'Étoile, France). Mock-infected C8166 cells were used as a negative control. In parallel, HIV-1_IIIb_-infected C8166 cells were treated with 5 µM tenofovir (NIBSC, London, UK) with the same procedure used for the drug treatment.

### RNA isolation and quantitative nucleic acid analysis

Total RNA was extracted using the NucleoSpin RNA kit (Macherey-Nagel, Düren, Germany), and 1 μg was retrotranscribed using the Revert-Aid H-Minus FirstStrand cDNA Synthesis Kit (Thermo Fisher Scientific, Waltham, USA), according to the manufacturer’s instructions. Comparison of mRNA expression between samples (i.e., infected vs. untreated) was performed by SYBR green-based RT-qPCR using Mx3000P apparatus (Stratagene, San Diego, USA), using the primers reported in Supplementary Table 1. To determine the number of viral DNA genomes per nanogram of cellular reference DNA (GAPDH gene), viral DNA levels were measured by quantitative qPCR on an Mx3000P apparatus (Stratagene, San Diego, USA). HCMV DNA copy numbers were normalized by dividing by the amount of human GAPDH gene amplified per reaction mixture. A standard curve of serially diluted genomic DNA mixed with an IE1-encoding plasmid (from 10^7^ to 1 copy) was created in parallel with each analysis.

### Western blot analysis

Whole-cell protein extracts were prepared and subjected to immunoblotting^53^. Briefly, equal amounts of cell extracts were fractionated by electrophoresis on SDS-polyacrylamide gels and transferred to Immobilon-P membranes (Merck Millipore, Burlington, MA, USA). After blocking with TBS (Tris-buffered saline containing 0.05% Tween20) containing 5% milk, membranes were incubated overnight at 4 °C with the appropriate primary antibodies. In this study, the primary antibodies used were: anti-peptidyl-citrulline, clone F95 (1:500), anti-IFIT1 (1:500), anti-V5 (1:1000), anti-pp65 (1:1000), anti-pp28 (1:1000), anti-UL44 (1:1000), anti-Mx1 (1:500), anti-IEA (1:1000), anti-PAD2 (1:1500), anti-PAD6 (1:500), anti-PAD4 (1:500), anti-PAD3 (1:500), anti-PAD1 (1:500), anti-actin clone C4 (1:1000), and anti-α-tubulin (1:1000). The primary antibodies used are detailed in Supplementary Table 2. Membranes were then washed and incubated for 1 h at room temperature with secondary antibodies: anti-rabbit/mouse IgG and anti-mouse IgM, horseradish peroxidase-linked species-specific whole antibody (Amersham, Merck Life Science S.r.l., Milan, Italy) or goat anti-mouse IgM antibody (Merck Life Science S.r.l., Milan, Italy) (1:2000). Proteins were visualized using ChemiDoc MP Imaging System (Bio-Rad Laboratories Srl), and an enhanced chemiluminescence detection kit (Thermo Fisher Scientific, Waltham, MA, Stati USA). Scanning densitometry of the bands was performed using Image Lab (version 4.6.9; Bio-Rad Laboratories S.r.l., Segrate, Italy).

### Detection of citrullination with rhodamine–phenylglyoxal (Rh–PG)

Equal amounts of protein were diluted with 80% trichloroacetic acid and incubated with Rh–PG (final concentration 0.1 mM) for 30 min^25^. The reaction was quenched with 100 mM l-citrulline, then centrifuged at 21,100×*g* for 10 min and washed with ice-cold acetone, and resuspended in 2× SDS loading dye for gel electrophoresis. Gels were imaged (excitation = 532 nm, emission = 580 nm) using a ChemiDoc MP Imaging System (Bio-Rad Laboratories Srl), stained with brilliant blue G-colloidal solution (Sigma-Aldrich, Milan, Italy).

### Enzyme-linked immunosorbent assay activity

Plates (Nunc^®^ 96 MaxiSorp™, Sigma-Aldrich, Milan, Italy) were coated with 4 μg/mL of calf thymus histone 3 (Sigma-Aldrich, Milan, Italy) in coating buffer (100 mM Na_2_CO_3_, 100 mM NaHCO_3_, pH 9.6) and incubated at 4 °C overnight. Subsequently, they were washed with 1× TBST (Tris-buffered saline, 0.05 % Tween20). Then, 50 µg of protein lysates from mock- or HCMV-infected HFFs at different time points, in the presence or absence of 100 µM Cl-A or with the same volume of the vehicle as a negative control, were diluted in calcium-free PAD reaction buffer (2 mM DTT, 50 mM NaCl,100 mM Tris, pH 7.57). As a positive control, increasing concentrations of 2.5, 5, and 10 mU of recombinant PAD2 (Cayman Chemical, Ann Arbor, USA), diluted in PAD reaction buffer containing 10 mM CaCl_2_, were applied. The samples were incubated for 20 h at 37 °C. Plates were blocked in 100 μL/well of blocking solution (2% BSA in 1× PBST) at room temperature for 1 h. After incubation with blocking buffer, an anti-human citrullinated histone 3 primary antibody (1:2000, ab5103; Abcam, Cambridge, UK) and anti-rabbit IgG-Fc HRP-conjugated secondary antibody were used to detect citrullinated proteins (1:7000). Subsequently, 100 µL of 3,3’,5,5’-tetramethylbenzidine (TMB) (Sigma-Aldrich, Milan, Italy) was added to each well. The amplification of the signal by HRP was given by the blue color obtained after 30 min of incubation, which changed to yellow once stopped by the addition of 1 N HCl. The optical density (OD) was measured at 450–620 nm using a Victor X3 Multilabel Reader (Perkin Elmer, Waltham, MA, USA; wallac 1420 work station software)^54,55^.

### Construction of promoter-reporter plasmids

The 5’-flanking region of *PADI2* was generated by PCR using Q5 High-Fidelity DNA polymerase (New England Biolabs, Ipswich, USA), pLightSwitch_Prom *PADI2* (Active Motif, La Hulpe, Belgium), as the template, and the primers listed in Supplementary Table 3. The thermocycler (Biorad C1000 Touch Thermal Cycler) settings consisted of 30 s of incubation at 98 °C, followed by 35 cycles at 98 °C for 10 s, 30 s at the predicted melting temperature and 30 s at 72 °C, and a final extension for 2 min at 72 °C. The 5’-flanking region of *PADI4* was amplified by PCR using human genomic DNA from HFFs, as the template, and the primers listed in Supplementary Table 2. The PCR condition was an initial denaturation for 2 min at 95 °C, 35 cycles (95 °C for 30 s, 56 °C for 30 s, and 72 °C for 4 min) and a final extension at 72 °C for 8 min. The resulting amplification products were digested with XhoI (Thermo Fisher Scientific, Waltham, USA) and HindIII (Thermo Fisher Scientific, Waltham, USA) and cloned into the pGL4.20[luc2/Puro] Vector (Promega, Madison, USA), which encodes the luciferase reporter gene luc2 (*Photinus pyralis*), but no regulatory elements. All of the constructs were prepared using the PureYield Plasmid Miniprep System (Promega, Madison, USA) and verified by restriction mapping and complete sequencing. The resulting chromatograms were analyzed using Chromas software 2.6.6 (Technolysium Ltd.).

### Luciferase assay

HFFs were electroporated using a Micro-Porator MP-100 (Thermo Fischer Scientific, Waltham, USA), according to the manufacturer’s instructions (a single 1300 V pulse, 30-ms pulse width). Briefly, 500 ng of each construct were used every 2 × 10^5^ cells, which were plated in 24-well tissue culture clusters at a density of 2 × 10^5^ cells/well. To correct for transfection efficiency, all cells were co-transfected with the pRL-SV40 vector (Promega, Madison, USA), which contained the Renilla luciferase gene driven by the SV40 promoter. After 24 h, cells were infected with HCMV, UV-HCMV, or mock (MOI of 1 PFU/ml). At 24 hpi, firefly and Renilla luciferase activities were measured using the Dual-Luciferase reporter assay system kit (Promega, Madison, USA) and a Victor X3 Multilabel Plate Reader (Perkin Elmer, Waltham, MA, USA; wallac 1420 work station software). Firefly luciferase activity from the luciferase reporter vector was normalized to the Renilla luciferase activity from the pRL-SV40 vector. Values were expressed as the ratio of relative light units (RLU) measured for firefly luciferase activity to the RLU measured for that of Renilla luciferase.

### CRISPR-Cas9 vector constructs

The CRISPR/Cas9 system was employed to generate specific gene knockouts in HFFs. Briefly, a lentiviral CRISPR/Cas9 vector that encodes a codon-optimized nuclear-localized Cas9 gene N-terminally fused to the puromycin resistance gene via a T2A ribosome-skipping sequence was employed. This vector contains a human U6 promoter driving expression of a guide RNA (gRNA) consisting of a gene-specific CRISPR RNA (crRNA) fused to the trans-activating crRNA (tracrRNA) and a terminator sequence^56^. gRNA sequences are reported in Supplementary Table 4. An empty vector carrying no gRNA was used as negative control (WT cell line). All constructs were verified by Sanger sequencing (Chromas 2.6.6).

### Lentivirus production and transduction of HFFs with lentiviral CRISPR/Cas9

Recombinant lentiviruses were packaged in HEK 293T cells by cotransfection of the 3rd Generation Packaging System Mix (kindly provided by A. Follenzi, University of Eastern Piedmont, Novara, Italy) with the above mentioned vectors to produce viral particles using Lipofectamine 2000 (Thermo Fisher Scientific, Waltham, USA). Viral supernatants were harvested after 72 h and used to transduce HFFs by infection in the presence of 8 μg/ml polybrene. Transduced cells were selected with puromycin (1 μg/ml) over the course of 14-day post transduction. After selection, successful knockout was confirmed using immunoblotting and TIDE analysis.

### TIDE

After selection, successful knockout was confirmed using immunoblotting. In addition, indel frequencies were quantified using TIDE^57^. Genomic DNA was extracted, and PCR amplicons spanning the single-guide RNA (sgRNA) target site were generated. The purified PCR products were then Sanger sequenced, and indel frequencies were quantified using the TIDE software (http://tide.nki.nl) (Supplementary Fig. 3). A reference sequence (wild-type cells) was used as a control. Genomic DNA was isolated from 1 × 10^6^ cells using the ISOLATE II Genomic DNA Kit (Bioline Meridian Biosciences, Paris, France). PCR reactions were carried out with 50 ng of genomic DNA and Q5 High-Fidelity DNA polymerase according to the manufacturer’s instructions. PCR conditions were 30 s at 98 °C (1 cycle), followed by 10 s at 98 °C, 30 s at 5 °C and 30 s at 72 °C (35 cycles) (Biorad C1000 thermocycler). The PCR products were purified using the GeneJET Gel Extraction and DNA Cleanup Micro Kit (Thermo Fischer Scientific, Waltham, USA). The primer pairs spanning the target site are reported in Supplementary Table 5.

### Pull down

Uninfected or HCMV-infected cells (MOI of 1 PFU/ml) were washed with 1× PBS and lysed in radioimmunoprecipitation assay (RIPA) buffer (50 mM Tris pH 7.4; 150 mM NaCl; 1 mM EDTA; 1% nonidet P-40; 0.1% SDS; 0.5% deoxycholate; protease inhibitors). Proteins (200 µg) were then incubated with 2 μg of F95 antibody or with an isotype antibody as negative control (62-6820; Thermo Fischer Scientific, Waltham, USA) for 1 h at room temperature with rotation followed by overnight incubation at 4 °C with protein G-Sepharose (Sigma-Aldrich, Milan, Italy). Immune complexes were collected by centrifugation and washed with RIPA buffer. The Sepharose beads were pelleted and washed three times with RIPA buffer, resuspended in reducing sample buffer (50 mM Tris pH 6.8; 10% glycerol; 2% SDS; 1% 2-mercaptoethanol), boiled for 5 min, and resolved on a SDS-PAGE gel to assess protein binding by immunoblotting.

### Citrullinome analysis by mass spectrometry: sample preparation

Sample preparation in technical triplicates followed the procedure outlined in ref. ^58^. Equal amounts of cell lysates from each experimental group (300 μg) were diluted in buffer (100 mM HEPES pH 7.6) to a final concentration of 1 μg/μL and incubated with 20% trichloroacetic acid (TCA) and 0.5 mM biotin-PG^59^ for 30 min at 37 °C. Labeled proteomes were precipitated on ice for 30 min. Samples were pelleted through tabletop centrifugation (21,100×*g*, 15 min) at 4 °C. The supernatants were discarded, and the pellets were washed with cold acetone (300 μL). After drying for 5 min, the pellets were resuspended in 1.2% SDS in PBS by bath sonication and heating. Samples were then transferred to 15-mL screw-cap tubes and diluted in 1× PBS to a 0.2% SDS final concentration. Samples were incubated with streptavidin agarose slurry (Sigma-Aldrich, 170 μL) overnight at 4 °C and for an additional 3 h at 25 °C. After discarding the flow-through, the streptavidin beads were washed with 0.2% SDS in PBS (5 mL) for 10 min at 25 °C. The beads were then washed three times with 1X PBS (5 mL) and three times with water (5 mL) in order to remove any unbound proteins. Beads were then transferred to a screw-cap microcentrifuge tube and heated in 1× PBS with 500 μL 6 M urea and 10 mM DTT (65 °C, 20 min). Proteins bound to the beads were then alkylated with iodoacetamide (20 mM, 37 °C for 30 min). The beads were successively pelleted by centrifugation (1400 ×*g* for 3 min) and the supernatant was removed. The pellet was resuspended in a premixed solution of 2 M urea, 1 mM CaCl_2_, and 2 μg Trypsin Gold (Promega, Madison, USA) in PBS. These were shaken overnight at 37 °C. The supernatant was collected and the beads were washed twice with water (50 μL), each time collecting the supernatant. The fractions were combined, acidified with formic acid (5% final concentration), and stored at −20 °C until use.

### Mass spectrometry

Liquid chromatography-mass spectrometry/mass spectrometry (LC-MS/MS) analysis was performed with an LTQ-Orbitrap Discovery mass spectrometer (Thermo Fisher Scientific, Waltham, MA, USA) coupled to an Easy-nLC HPLC (Thermo Fisher Scientific, Waltham, MA, USA). Samples were pressure-loaded onto a 250-µm fused-silica capillary hand-packed with 4 cm Aqua C18 reverse-phase resin (Phenomenex). Samples were separated on a hand-packed 100-µm fused-silica capillary column with a 5-µm tip packed with 10 cm Aqua C18 reverse-phase resin (Phenomenex). Peptides were eluted using a 10-h gradient of 0–100% Buffer B in Buffer A (Buffer A: 95% water, 5% acetonitrile, 0.1% formic acid; Buffer B: 20% water, 80% acetonitrile, 0.1% formic acid). The flow rate through the column was set to ~400 nL/min, and the spray voltage was set to 2.5 kV. One full MS scan (FTMS) was followed by seven data-dependent MS2 scans (ITMS) of the *n*th most abundant ions. The tandem MS data were searched by the SEQUEST algorithm using a concatenated target/decoy variant of the human and viral UniProt database. A static modification of +57.02146 on cysteine was specified to account for alkylation by iodoacetamide. SEQUEST output files were filtered using DTASelect 2.0.

### Reductive dimethylation (ReDiMe) labeling and mass spectrometry for the citrullinome analysis of wtAD169 and AD169ΔIE1-infected cells

The trypsin digest was desalted using Pierce C18 spin column (catalog No 89870) according to the manufacturer’s protocol and was resuspended in 200 µL of 100 mM triethylammonium bicarbonate, pH 8.5. 20% H^12^CHO (4 µL, light formaldehyde), D^12^CDO (4 µL, medium formaldehyde), and D^13^CDO (4 µL, heavy formaldehyde) were added to the mock, AD169- and AD169∆IE1-infected samples, respectively. In total, 20 µL of 0.6 M sodium cyanoborohydride (NaBH_3_CN) was then added to both the mock and AD169-infected samples, respectively, while 20 µL of 0.6 M sodium cyanoborodeuteride (NaBD_3_CN) was added to the AD169∆IE1-infected samples. The samples were incubated at room temperature for 2 h. The samples were then cooled on ice and the reaction quenched with 4 µL of 20% ammonium hydroxide. Formic acid (8 µL) was then added to the samples. Heavy, medium, and light formaldehyde-labeled samples were mixed together, and the mixture was desalted and stored at −20 °C for proteomic analysis. Data was acquired using a NanoAcquity UPLC (Waters Corporation, Milford, MA) coupled to an Orbitrap Fusion Lumos Tribrid (Thermo Fisher Scientific, Waltham, MA) mass spectrometer. Peptides were trapped and separated using an in-house 100 µm I.D. fused-silica pre-column (Kasil frit) packed with 2 cm ProntoSil (Bischoff Chromatography, DE) C18 AQ (200 Å, 5 µm) media and configured to an in-house packed 75 µm I.D. fused-silica analytical column (gravity-pulled tip) packed with 25 cm Magic (Bruker, Billerica, MA) C18-AQ (100 Å, 3 µm) media, respectively. Mobile phase A was water supplemented with 0.1 % (v/v) formic acid, and mobile phase B was acetonitrile supplemented with 0.1 % (v/v) formic acid. Following a 3.8 µL of sample injection, peptides were trapped at flow rate of 4 µL/min with 5% B for 4 min, followed by gradient elution at a flow rate of 300 nL/min from 5 to 35% B over 120 min (total run time 145 min). Electrospray voltage was delivered by a liquid junction electrode (1.5 kV) located between the columns and the transfer capillary to the mass spectrometer was maintained at 275 °C. Mass spectra were acquired over *m/z* 375–1500 Da with a resolution of 120,000 (*m/z* 200), a maximum injection time of 110 ms, and an AGC target of 400,000. Tandem mass spectra were acquired using data-dependent acquisition (3 s cycle) with an isolation width of 1.6 Da, HCD collision energy of 30%, resolution of 15,000 (*m/z* 200), maximum injection time of 50 ms, and an AGC target of 50,000.

### Database search

Raw data were processed and searched using Maxquant 1.6.14 and its integrated Andromeda search engine using the Swiss-Prot human (downloaded 04/09/2019) and Uniprot HCMV (downloaded 02/27/2021). Search parameters were as follows: tryptic digestion with up to 2 missed cleavages; peptide N-terminal acetylation, methionine oxidation, N-terminal glutamine to pyroglutamate conversion were specified as variable modifications. The monoisotopic mass increment of the triplex dimethyl labels, light, medium, and heavy dimethyl labels at 28.0313, 32.0564, and 36.0757 Da, respectively, were set as a variable modification on the peptide N-termini and lysine residues. Carbamidomethylation of cysteines was set as static modification. The main search tolerance was 6 ppm, and the first search tolerance was 50 ppm. Both the protein and peptides identification false discovery rates (FDR) were <1%. Protein grouping, dimethyl ratio calculations, and downstream statistics were performed in Scaffold Q + S 4.8.9 (Proteome Software, Portland, OR).

### siRNA-mediated knockdown

HFFs were transiently transfected with a Micro-Porator (Digital Bio Pharm, London, Great Britain) according to the manufacturer’s instructions (1200 V, 30 ms pulse width, one impulse) with a pool of small interfering RNAs (Qiagen, Hilden, Germany) targeting Mx1 (siMx1, FlexiTube siRNAs cat. Nos.: SI02781093, SI05459538, SI04435963, SI04435956), PAD2 (siPAD2, FlexiTube siRNAs cat. Nos.: SI04278953, SI04255860, SI04188793, SI00676872), PAD4 (siPAD4, FlexiTube siRNAs cat. Nos.: SI04939032, SI04359530, SI04299288, SI00676907) or control siRNA (siCTRL, 1027292) as a negative control. For siRNA-mediated knockdown of IFIT1, siRNA approach was performed according to Pichlmair et al. (siIFIT1_1: CTCCTTGGGTCGTTCTACAAA; siIFIT1_2: TACATGGGAGTTATCCATTGA)^34^.

### Recombinant adenoviral vector production

The adenovirus-derived vectors (AdVs) expressing IFIT1 and LacZ were generated by means of a replacement strategy using recombineering methods, as described previously^60,61^. The open reading frame (ORF) was amplified using a specific set of primers for each desired construct (Supplementary Table 6), and colonies were analyzed by PCR and sequencing (Supplementary Table 6). IFIT1 and LacZ expression was assessed by western blotting using a V5 antibody. For cell transduction, HFFs were incubated with AdVIFIT1 at an MOI of 10 in DMEM. After 2 h at 37 °C, the virus was washed off, and fresh medium was applied. At 24 hpt, HFFs were infected with HCMV strain Merlin at an MOI of 1 and incubated for 144 h.

### UL54 and UL44 production and in vitro citrullination

Glutathione-S-transferase (GST) tagged UL54 was produced as described^62^ and was kindly provided by Han Chen. UL44∆C290 was produced as described^63^ with several modifications: after resuspension, cells were lysed by sonication at an amplitude of 40% for a total of ~15 min, pulsing in cycles of 5 s on and 9 s off. The lysate was centrifuged at 18,000×*g* for 1.5 h at 4 °C, then applied to a glutathione-Sepharose 4 FastFlow column (GE Healthcare) that had been equilibrated in buffer A (50 mM Tris-HCl (pH 7.5), 10 mM EDTA, 2 mM dithiothreitol (DTT), 500 mM NaCl, 20% glycerol), and then washed extensively with the same buffer. UL44∆C290 was eluted with buffer B (Buffer A + 15 mM glutathione), and fractions containing the protein were combined and diluted 1.5:1 with buffer C (50 mM Tris-HCl [pH 7.5], 1 mM EDTA, 2 mM DTT, 10% glycerol). To cleave off the GST tag, the sample was treated with HRV 3C protease (Takara) overnight at 4 °C, loaded onto a heparin agarose column (Protein Ark), and eluted with a linear gradient of 150–1000 mM NaCl in buffer C. Fractions containing UL44∆C290 were combined and applied to a HiLoad^®^ Superdex^®^ 200 column (GE Healthcare) in storage buffer (50 mM Tris-HCl (pH 7.4), 0.1 mM EDTA, 2 mM DTT, 500 mM NaCl, 20% Glycerol). Fractions containing UL44∆C290 were combined, concentrated using an Amicon^®^ Ultra-15 Centrifugal Filter Device (Merck Millipore, Milan), and stored in aliquots at −80 °C.

In vitro citrullination of UL44 was performed as follows: 6 μM UL44∆C290 was incubated in PAD assay buffer (50 mM HEPES, pH 7.6, 10 mM CaCl_2_, 150 mM NaCl, 2 mM DTT, 12.5% glycerol for UL44) with recombinant human PAD2 or PAD4 (Chemical Cayman) at a concentration of 0.7 µM for 180 min, at 37 °C. Citrullination was terminated by the addition of 0.05 M EDTA. As a control, a reaction was performed without PAD enzymes (mock treatment). Samples were purified on a Superose 6 Increase GL column (GE Healthcare) in 50 mM Tris-HCl [pH 7.5], 1 mM EDTA, 2 mM DTT, 150 mM NaCl, 10% glycerol, to remove the vast majority of the PAD enzymes. Fractions from each reaction containing UL44∆C290 were concentrated using an Amicon^®^ Ultra-15 Centrifugal Filter Device (Merck Millipore, Milan), and stored at −20 °C. In order to estimate the amount of residual PAD enzymes, 2.2 μg of each UL44∆C290 sample was applied to SDS-PAGE, stained with Coomassie brilliant blue, and bands corresponding to PAD2 or PAD4 contamination were quantified relative to known amounts of PAD2 and PAD4 (Image Lab).

### Production of recombinant human IFIT1 and in vitro citrullination

Recombinant IFIT proteins were expressed in *E. coli* and were purified on a HisTrap HP column (GE Healthcare, Chicago, IL, Stati Uniti).

The in vitro citrullination of IFIT1 was performed as previously described for UL44.

### LC-MS/MS analysis of mock- or PAD2-treated recombinant IFIT1

One μg of recombinant IFIT1, mock or PAD2-treated, was digested with 1:50 LysC (Wako #129-02541) overnight at 37 °C. Peptides were purified and concentrated on stage tips with three C18 Empore filter discs (3 M), and further measured via LC-MS/MS, using an EASY- nLC 1200 system (Thermo Fisher Scientific, Waltham, MA, USA) coupled to a Q-Exactive HF mass spectrometer (Thermo Fisher Scientific, Waltham, MA, USA), on a 20-cm reverse-phase analytical column (75 μm column diameter; ReproSil-Pur C18-AQ 1.9-μm resin; Dr. Maisch) and separated using a 60 min acetonitrile gradient. Raw files were processed using MaxQuant version 1.6.0.15 with carbamidomethyl (Cys) as fixed modification as well as oxidation (Met), Acetyl (Protein N-term) and citrullination (R), as defined by H(-1) N(-1) O, as variable modifications.

### In vitro transcription 7SK 5’-PPP-RNA and gel shift assay

Non-coding RNA (7SK anti-sense 356 bp) was transcribed in vitro using the SP6 MEGAscript kit (Thermo Fisher Scientific, Waltham, MA, USA)) as per the manufacturer’s instruction^64^. In all, 50 nM (5 pmol) of in vitro transcribed 7SK 5’-ppp-RNA was incubated with 5 µM of recombinant IFIT1, either mock or treated with PAD2 for 3 h, for 30 min at room temperature in PBS—400 mM DTT—100 mM NaCl, and loaded on a 0.8% Tris-Glycine agarose gel. The gel was stained in a 3× Gel Red (Biotium, #41003) solution before imaging.

### Functional assay of UL44

Long-chain DNA synthesis by UL54 and UL44 on a poly(dA)-oligo(dT)_12-18_ primer template (Amersham Biosciences, Little Chalfont, UK) with radiolabeled [^32^P]dTTP (~55 Ci/mmol) as a substrate was measured using a modification of a gel-based assay described previously^38^. Reactions contained 50 mM TRIS-HCl [pH 7.5], 100 mM (NH_4_)_2_SO_4_, 3 mM MgCl_2_, 0.1 mM EDTA, 1 mM DTT, 4 % Glycerol, 40 µg/mL BSA, and using 26.5 nM GST-UL54 alone or with 58 nM of untreated UL44∆C290 (UNT control), or with PAD2-treated UL44∆C290, PAD4-treated UL44∆C290, or mock-treated UL44∆C290. To control for any effects of trace amounts of PAD enzymes contaminating UL44∆C290 purified from citrullination reactions, in separate mixtures UNT control reactions were supplemented with 270 pM PAD2 or 370 pM PAD4. Gels with incorporated radioactivity were exposed to phosphorescence screens that were scanned with a Typhoon scanner (Amersham Biosciences, Little Chalfont, UK) and quantified using Image Lab. Data were normalized in each experiment by setting the UNT control as 100%. Statistical analysis was performed by one-way analysis of variance (ANOVA) with Dunnett’s correction using GraphPad Prism version 8.4.3 for Windows.

### GO protein class

PANTHER (http://www.pantherdb.org) gene list analysis was used to functionally classify citrullinated proteins based on protein classes based on the UniProt ID code^65^.

### Statistical analysis

All statistical tests and the IC50 were performed using GraphPad Prism version 5.00 or 8.4.3 for Windows (GraphPad Software, San Diego, USA). The data were presented as means ± standard error of mean (SEM). Statistical significance was determined by using an unpaired *t* test (two-tailed), one-way or two-way analysis of variance (ANOVA) with Bonferroni’s, or Dunnett’s post tests. Differences were considered statistically significant for *P* < 0.05 (*P < 0.05; **P < 0.01; ***P < 0.001).

### Reporting summary

Further information on research design is available in the Nature Research Reporting Summary linked to this article.

## Supplementary information

Supplementary Information

Description of Additional Supplementary Files

Supplementary Data 1

Supplementary Data 2

Supplementary Data 3

Supplementary Data 4

Supplementary Data 5

Supplementary Data 6

Reporting Summary

## Data Availability

The data that support the findings of this study are available within the paper and its supplementary information files. All other relevant data are available from the corresponding authors upon reasonable request. The mass spectrometry proteomics data have been deposited to the ProteomeXchange Consortium via the PRIDE [1] partner repository with the dataset identifier PXD025803, and PXD025818. Source data are provided with this paper.

## Source data

Source Data

## References

[1] Griffiths P, Baraniak I, Reeves M (2015). The pathogenesis of human cytomegalovirus. J. Pathol..

[2] Britt WJ (2017). Congenital human cytomegalovirus infection and the enigma of maternal immunity. J. Virol..

[3] Halenius A, Hengel H (2014). Human cytomegalovirus and autoimmune disease. Biomed. Res. Int..

[4] Soderberg-Naucler C (2006). Does cytomegalovirus play a causative role in the development of various inflammatory diseases and cancer?. J. Intern. Med..

[5] Du Y, Zhang G, Liu Z (2018). Human cytomegalovirus infection and coronary heart disease: a systematic review. Virol. J..

[6] Jackson SE (2017). CMV immune evasion and manipulation of the immune system with aging. GeroScience.

[7] Cobbs CS (2014). Does valganciclovir have a role in glioblastoma therapy?. Neuro. Oncol..

[8] Herbein, G. The human cytomegalovirus, from oncomodulation to oncogenesis. *Viruses* **10**, 408 (2018). 10.3390/v10080408 PMC6115842 30081496

[9] Baker PJ (2017). Posttranslational modification as a critical determinant of cytoplasmic innate immune recognition. Physiol. Rev..

[10] Gudmann NS, Hansen NUB, Jensen ACB, Karsdal MA, Siebuhr AS (2015). Biological relevance of citrullinations: diagnostic, prognostic and therapeutic options. Autoimmunity.

[11] Bicker KL, Thompson PR (2013). The protein arginine deiminases: structure, function, inhibition, and disease. Biopolymers.

[12] van Venrooij WJ, van Beers JJBC, Pruijn GJM (2011). Anti-CCP antibodies: the past, the present and the future. Nat. Rev. Rheumatol..

[13] Vossenaar ER, van Venrooij WJ (2004). Citrullinated proteins: sparks that may ignite the fire in rheumatoid arthritis. Arthritis Res. Ther..

[14] Knight JS (2015). Peptidylarginine deiminase inhibition disrupts NET formation and protects against kidney, skin and vascular disease in lupus-prone MRL/ *lpr* mice. Ann. Rheum. Dis..

[15] Acharya NK (2012). Neuronal PAD4 expression and protein citrullination: possible role in production of autoantibodies associated with neurodegenerative disease. J. Autoimmun..

[16] Yang L, Tan D, Piao H (2016). Myelin basic protein citrullination in multiple sclerosis: a potential therapeutic target for the pathology. Neurochem. Res..

[17] Nicholas AP (2011). Dual immunofluorescence study of citrullinated proteins in Parkinson diseased substantia nigra. Neurosci. Lett..

[18] Yuzhalin AE (2019). Citrullination in cancer. Cancer Res..

[19] Sokolove J (2013). Brief report: citrullination within the atherosclerotic plaque: a potential target for the anti-citrullinated protein antibody response in rheumatoid arthritis. Arthritis Rheum..

[20] Casanova, V. et al. Citrullination alters the antiviral and immunomodulatory activities of the human cathelicidin LL-37 during rhinovirus infection. *Front. Immunol*. **11**, 85 (2020). 10.3389/fimmu.2020.00085 PMC7010803 32117246

[21] Trier NH (2018). Antibodies to a strain-specific citrullinated Epstein-Barr virus peptide diagnoses rheumatoid arthritis. Sci. Rep..

[22] Pratesi F (2011). Antibodies to a new viral citrullinated peptide, VCP2: fine specificity and correlation with anti-cyclic citrullinated peptide (CCP) and anti-VCP1 antibodies. Clin. Exp. Immunol..

[23] Pratesi F, Tommasi C, Anzilotti C, Chimenti D, Migliorini P (2006). Deiminated Epstein-Barr virus nuclear antigen 1 is a target of anti–citrullinated protein antibodies in rheumatoid arthritis. Arthritis Rheum..

[24] Arisan ED, Uysal-Onganer P, Lange S (2020). Putative roles for peptidylarginine deiminases in COVID-19. Int. J. Mol. Sci..

[25] Bicker KL, Subramanian V, Chumanevich AA, Hofseth LJ, Thompson PR (2012). Seeing citrulline: development of a phenylglyoxal-based probe to visualize protein citrullination. J. Am. Chem. Soc..

[26] Scherer M (2016). Characterization of recombinant human cytomegaloviruses encoding IE1 mutants L174P and 1-382 reveals that viral targeting of PML bodies perturbs both intrinsic and innate immune responses. J. Virol..

[27] Luo Y, Knuckley B, Lee Y-H, Stallcup MR, Thompson PR (2006). A fluoroacetamidine-based inactivator of protein arginine deiminase 4: design, synthesis, and in vitro and in vivo evaluation. J. Am. Chem. Soc..

[28] Fensterl, V. & Sen, G. C. Interferon-induced ifit proteins: their role in viral pathogenesis. 10.1128/JVI.02744-14 (2015). 10.1128/JVI.02744-14 PMC4325746 25428874

[29] Li D, Swaminathan S (2019). Human IFIT proteins inhibit lytic replication of KSHV: a new feed-forward loop in the innate immune system. PLoS Pathog..

[30] Zhang L (2017). Antiviral effects of IFIT1 in human cytomegalovirus-infected fetal astrocytes. J. Med. Virol..

[31] Haller O, Staeheli P, Schwemmle M, Kochs G (2015). Mx GTPases: dynamin-like antiviral machines of innate immunity. Trends Microbiol..

[32] Schilling, M. et al. Human MxB protein is a pan-herpesvirus restriction factor. *J. Virol*. **92**, JVI.01056-18 (2018). 10.1128/JVI.01056-18 PMC6096802 29950411

[33] Crameri M (2018). MxB is an interferon-induced restriction factor of human herpesviruses. Nat. Commun..

[34] Pichlmair A (2011). IFIT1 is an antiviral protein that recognizes 5′-triphosphate RNA. Nat. Immunol..

[35] Abbas YM (2017). Structure of human IFIT1 with capped RNA reveals adaptable mRNA binding and mechanisms for sensing N1 and N2 ribose 2’-O methylations. Proc. Natl Acad. Sci. USA.

[36] Ertl PF, Powell KL (1992). Physical and functional interaction of human cytomegalovirus DNA polymerase and its accessory protein (ICP36) expressed in insect cells. J. Virol..

[37] Weiland KL, Oien NL, Homa F, Wathen MW (1994). Functional analysis of human cytomegalovirus polymerase accessory protein. Virus Res..

[38] Loregian, A., Appleton, B. A., Hogle, J. M. & Coen, D. M. Residues of human cytomegalovirus DNA polymerase catalytic subunit UL54 that are necessary and sufficient for interaction with the accessory protein UL44. *J. Virol*. **78**, 158–167 (2004). 10.1128/JVI.78.1.158-167.2004 PMC303418 14671097

[39] Shelef MA, Bennin DA, Mosher DF, Huttenlocher A (2012). Citrullination of fibronectin modulates synovial fibroblast behavior. Arthritis Res. Ther..

[40] Tarcsa E (1996). Protein unfolding by peptidylarginine deiminase: Substrate specificity and structural relationships of the natural substrates trichohyalin and filaggrin. J. Biol. Chem..

[41] Barlow PG, Findlay EG, Currie SM, Davidson DJ (2014). Antiviral potential of cathelicidins. Future Microbiol..

[42] Barlow, P. G. et al. Antiviral activity and increased host defense against influenza infection elicited by the human cathelicidin LL-37. *PLoS ONE* **6**, e25333 (2011). 10.1371/journal.pone.0025333 PMC3198734 22031815

[43] Currie SM (2013). The human cathelicidin LL-37 has antiviral activity against respiratory syncytial virus. PLoS ONE.

[44] Wang G, Mishra B, Epand RF, Epand RM (2014). High-quality 3D structures shine light on antibacterial, anti-biofilm and antiviral activities of human cathelicidin LL-37 and its fragments. Biochimica et. Biophysica Acta—Biomembranes.

[45] Martineau AR (2007). IFN-γ- and TNF-independent vitamin D-inducible human suppression of mycobacteria: the role of cathelicidin LL-37. J. Immunol..

[46] Kilsgård O (2012). Peptidylarginine deiminases present in the airways during tobacco smoking and inflammation can citrullinate the host defense peptide LL-37, resulting in altered activities. Am. J. Respir. Cell Mol. Biol..

[47] Gariano GR (2012). The intracellular DNA sensor IFI16 gene acts as restriction factor for human cytomegalovirus replication. PLoS Pathog..

[48] Luganini A, Caposio P, Landolfo S, Gribaudo G (2008). Phosphorothioate-modified oligodeoxynucleotides inhibit human cytomegalovirus replication by blocking virus entry. Antimicrob. Agents Chemother..

[49] Bon I (2013). Peptide-derivatized SB105-A10 dendrimer inhibits the infectivity of R5 and X4 HIV-1 strains in primary PBMCs and cervicovaginal histocultures. PLoS ONE.

[50] Pignoloni B (2016). Distinct roles for human cytomegalovirus immediate early proteins IE1 and IE2 in the transcriptional regulation of MICA and PVR/CD155 expression. J. Immunol..

[51] E, X. et al. An E2F1-Mediated DNA damage response contributes to the replication of human cytomegalovirus. *PLoS Pathog*. **7**, e1001342 (2011). 10.1371/journal.ppat.1001342 PMC3093362 21589897

[52] Pauwels R (1988). Rapid and automated tetrazolium-based colorimetric assay for the detection of anti-HIV compounds. J. Virol. Methods.

[53] Dell’Oste V (2014). Innate nuclear sensor IFI16 translocates into the cytoplasm during the early stage of in vitro human cytomegalovirus infection and is entrapped in the egressing virions during the late stage. J. Virol..

[54] Zendman AJW (2007). ABAP: antibody-based assay for peptidylarginine deiminase activity. Anal. Biochem..

[55] Damgaard D, Senolt L, Nielsen MF, Pruijn GJ, Nielsen CH (2014). Demonstration of extracellular peptidylarginine deiminase (PAD) activity in synovial fluid of patients with rheumatoid arthritis using a novel assay for citrullination of fibrinogen. Arthritis Res. Ther..

[56] van de Weijer ML (2014). A high-coverage shRNA screen identifies TMEM129 as an E3 ligase involved in ER-associated protein degradation. Nat. Commun..

[57] Brinkman EK, Chen T, Amendola M, van Steensel B (2014). Easy quantitative assessment of genome editing by sequence trace decomposition. Nucleic Acids Res..

[58] Tilvawala R (2018). The rheumatoid arthritis-associated citrullinome. Cell Chem. Biol..

[59] Lewallen DM (2015). Chemical proteomic platform to identify citrullinated proteins. ACS Chem. Biol..

[60] Biolatti, M. et al. Human cytomegalovirus tegument protein pp65 (pUL83) dampens type I interferon production by inactivating the DNA sensor cGAS without affecting STING. *J. Virol*. **92**, e01774-17 (2018). 10.1128/JVI.01774-17 PMC5827387 29263269

[61] Chartier C (1996). Efficient generation of recombinant adenovirus vectors by homologous recombination in *Escherichia coli*. J. Virol..

[62] Chen H, Beardsley GP, Coen DM (2014). Mechanism of ganciclovir-induced chain termination revealed by resistant viral polymerase mutants with reduced exonuclease activity. Proc. Natl Acad. Sci. USA.

[63] Appleton BA, Loregian A, Filman DJ, Coen DM, Hogle JM (2004). The cytomegalovirus DNA polymerase subunit UL44 forms a C clamp-shaped dimer. Mol. Cell.

[64] Pichlmair A (2006). RIG-I-mediated antiviral responses to single-stranded RNA bearing 5′-phosphates. Science.

[65] Mi H, Muruganujan A, Ebert D, Huang X, Thomas PD (2019). PANTHER version 14: more genomes, a new PANTHER GO-slim and improvements in enrichment analysis tools. Nucleic Acids Res..

