# Supplementary material for: Human cytomegalovirus-induced host protein citrullination is crucial for viral replication
Source: Nat Commun. 2021 Jun 23;12:3910. doi: 10.1038/s41467-021-24178-6 (PMC8222335; doi:10.1038/s41467-021-24178-6)
Supplement: Supplementary file 1 — Supplementary Information [file 41467_2021_24178_MOESM1_ESM.pdf]

SUPPLEMENTARY INFORMATION

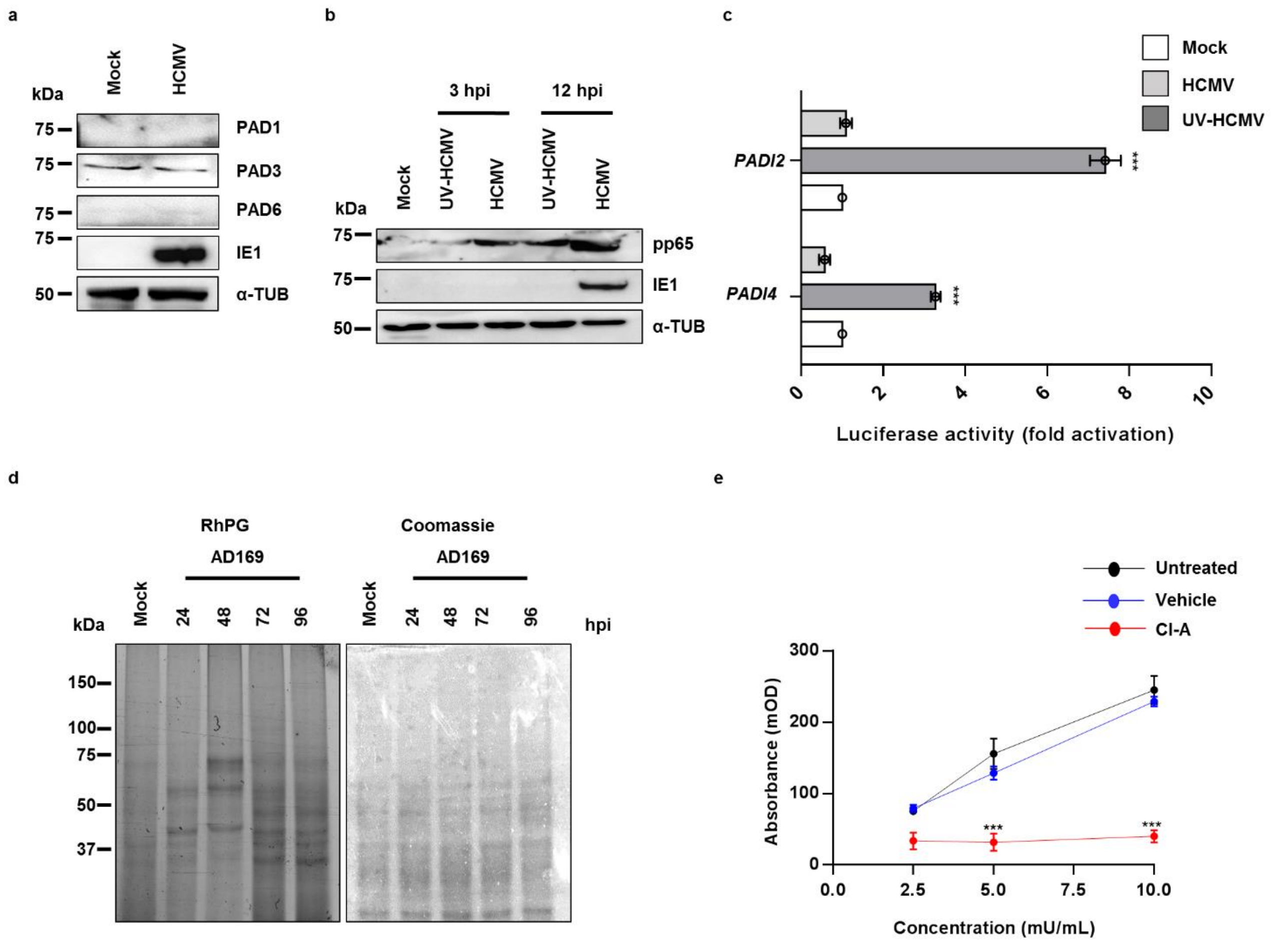

Supplementary Fig. 1. **PAD1, 3 and 6 protein expression during HCMV infection. a,** Protein lysates from uninfected (Mock) or infected HFFs at 48 hpi were subjected to immunoblotting using antibodies against PAD1, PAD3, PAD6, IEA (recognizing IE1-72- and IE2-86 kDa), or α-tubulin (α-TUB). The Western blot shown is representative of three independent experiments. **b,** UV-inactivated HCMV does not replicate. Protein lysates from uninfected (Mock) or infected HFFs (at 3 and 12 hpi) with either wild-type (HCMV) or UV-inactivated HCMV (UV-HCMV) were subjected to immunoblotting using antibodies against IEA (recognizing IE1-72 and IE2-86 kDa), pp65 or α-tubulin (α-TUB). **c,** *PADI2* and *PADI4* promoter activation upon HCMV infection. HFFs were transiently electroporated with luciferase plasmids encoding the wild-type *PADI2* or *PADI4* promoter region. Twenty-four h later, the cells were Mock-infected or infected with HCMV or UV-inactivated HCMV at an MOI of 1. At 24 hpi, firefly and Renilla luciferase activities were measured. Luciferase

activity in whole-cell lysates was normalized to Renilla luciferase activity and expressed as relative light units (RLU). Results are shown as a mean of fold change± SEM (error bars) of three independent experiments (*PADI2* promoter: Mock *vs.* HCMV $P < 0.001$; *PADI4* promoter: Mock *vs.* HCMV $P < 0.001$, two-way ANOVA followed by Bonferroni's post-tests). **d,** Protein lysates from HFFs infected with the HCMV strain AD169 (AD169) (MOI 1 PFU/cell) at 24, 48,72 and 96 h post infection (hpi) or from uninfected HFFs (Mock) were exposed to an Rh-PG citrulline-specific probe (left panel) and subjected to gel electrophoresis to detect citrullinated proteins. Equal loading was assessed by Coomassie blue staining (right panel). One representative gel of three independent experiments is shown. **e,** PAD enzymatic activity assay. Histone H3 was immobilized on a 96-well microtiter plate and incubated with increasing concentration of recombinant human PAD2 in the presence (red line) or absence (untreated or vehicle alone, black and blue line, respectively) of Cl-amidine. The conversion of peptidylarginine to peptidylcitrulline was detected by means of an anti-H3 citrulline antibody. Bound antibodies were detected and visualized by ELISA. Values are expressed as means ± SEM (error bars) of three independent experiments (5mU/mL rPAD2: Untreated *vs.* Cl-A $P < 0.001$; 10 mU/mL rPAD2: Untreated *vs.* Cl-A $P < 0.001$, two-way ANOVA followed by Bonferroni's post-tests). Data are shown as the mean ± SEM, *$P < 0.05$, **$P < 0.01$, ***$P < 0.001$.

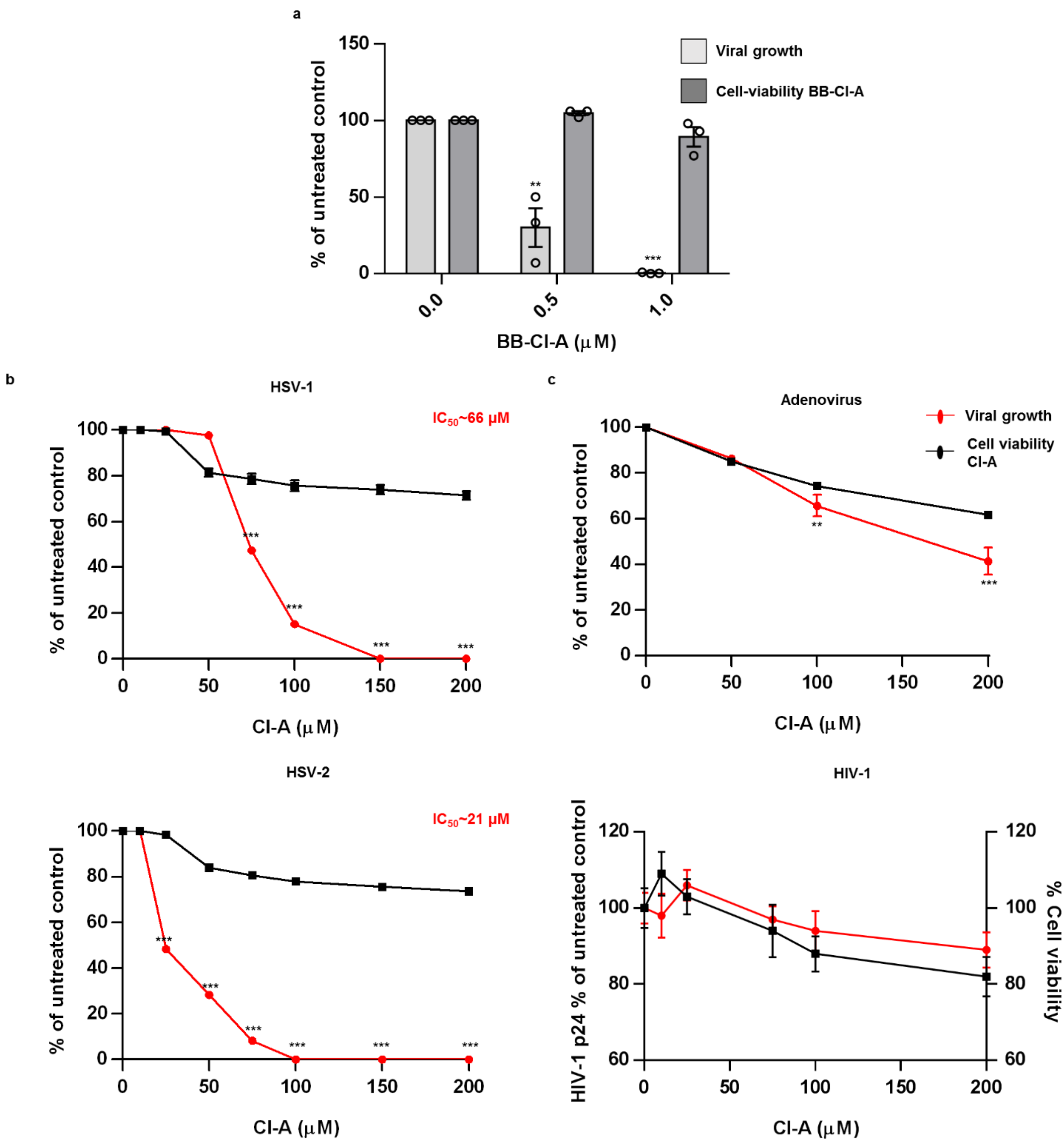

Supplementary Fig. 2. Antiviral activity of Cl-A against HSV-1 and HSV-2 replication. a, HFFs were infected with HCMV strain Merlin (MOI=0.1) and then treated with increasing concentrations of BB-Cl-A, which were given 1 h prior to virus exposure and kept throughout the whole experiment. At 144 hpi, viral plaques were microscopically counted, and the mean plaque count for each drug concentration was expressed as the percentage relative to the mean count of untreated controls. The number of plaques is plotted as a function of BB-Cl-A concentration. To determine cell viability, the

number of viable cells for each BB-Cl-A concentration was determined using MTT assay. Values are expressed as means ± SEM (error bars) of three independent experiments (Untreated *vs.* 0.5µM BB-Cl-A P=0.0014, Untreated *vs.* 1µM BB-Cl-A P< 0.001, one-way ANOVA followed by Bonferroni's post-tests). **b,** VERO cells were infected with HSV-1 (upper panel) or HSV-2 (lower panel) (MOI=0.1) and then treated with increasing concentrations of Cl-A, which were given 1 h prior to virus adsorption and kept throughout the whole experiment. At 48 hpi, viral plaques were microscopically counted and the mean plaque count for each drug concentration was expressed as percentage relative to the mean count of the untreated control. The number of plaques is plotted as a function of Cl-A concentration. The concentrations achieving 50% plaque formation (IC_{50}) reduction are shown (red line). To determine cell viability, the number of viable cells for each Cl-A concentration was determined using the MTT assay (black line). Values are expressed as means ± SEM (error bars) of three independent experiments (HSV-1: Untreated *vs.* 75 µM Cl-A P< 0.001, Untreated *vs.* 100 µM Cl-A P< 0.001, Untreated *vs.* 150 µM Cl-A P< 0.001, Untreated *vs.* 200 µM Cl-A P< 0.001; HSV-2: Untreated *vs.* 25 µM Cl-A P< 0.001, Untreated *vs.* 50 µM Cl-A P< 0.001, Untreated *vs.* 75 µM Cl-A P< 0.001, Untreated *vs.* 100 µM Cl-A P< 0.001, Untreated *vs.* 150 µM Cl-A P< 0.001, Untreated *vs.* 200 µM Cl-A P< 0.001, one-way ANOVA followed by Bonferroni's post test). **c,** (upper panel) HEK 293 cells were infected with a clinical isolate of adenovirus (MOI=0.1) and then treated with increasing concentrations of Cl-A as described above. At 144 hpi, viral plaques were microscopically counted, and the mean plaque count for each drug concentration was expressed as percentage of the mean count relative to the untreated control. The number of plaques is plotted as a function of Cl-A concentration (red line). To determine cell viability, the number of viable cells for each Cl-A concentration was determined using the MTT assay (black line). Values are expressed as means ± SEM (error bars) of three independent experiments. (lower panel) HIV-1_{IIIb} strain (5 ng/ml of HIV-1 gag p24) was incubated for 1 h at 37°C in RPMI 1640 medium with increasing concentrations of Cl-A. Successively, these Cl-A-containing solutions were used to grow C8166 lymphoblastoid $CD4^+$ T cells for 2 h at 37°C. HIV-1 replication was analyzed by HIV-1 p24 ELISA

assay on cell culture supernatants at day 7 post-infection. Values are expressed as the means ± SEM (error bars) of the level of HIV-1 gag p24 relative to untreated controls (set to 100%). Three independent experiments in duplicate were performed (red line). To determine cell viability, the number of viable cells for each Cl-A concentration was determined using trypan blue exclusion. Values are expressed as means ± SEM of viable cells relative to untreated controls (set to 100%) obtained from three independent experiments each performed in duplicate (black line) (AdV: Untreated *vs.* 100 µM Cl-A $P=0.0013$, Untreated *vs.* 200 µM Cl-A $P< 0.001$, one-way ANOVA followed by Bonferroni's post test). Data are shown as the mean ± SEM, *$P < 0.05$, **$P < 0.01$, ***$P < 0.001$.

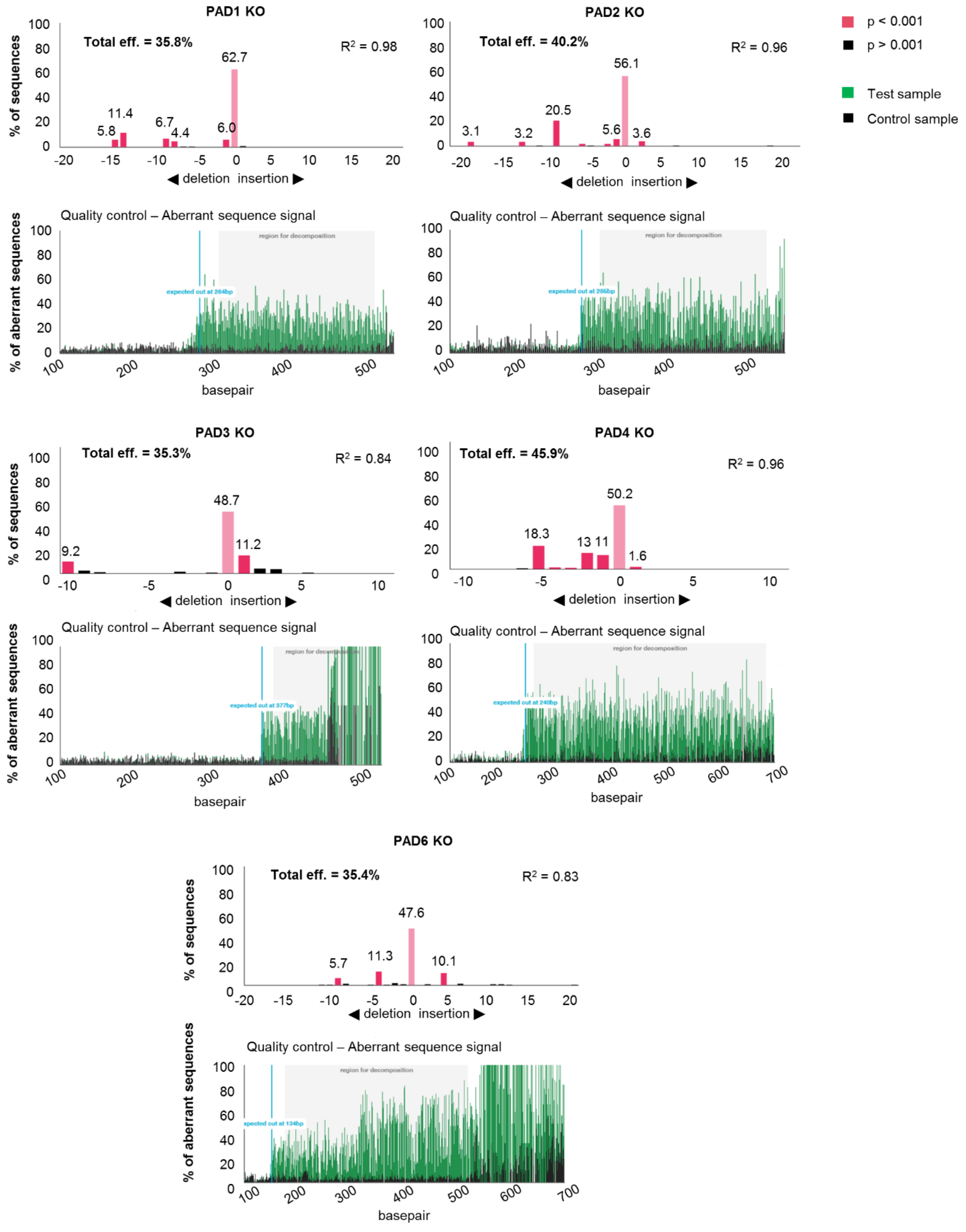

Supplementary Fig. 3. Assessment of genome editing in PAD1, PAD2, PAD3, PAD4, PAD6 KO cell lines. Successful genome editing was assessed by TIDE analysis. To this end, genomic DNA was extracted, and PCR amplicons spanning the single guide RNA (sgRNA) target site were generated.

The purified PCR products were then Sanger sequenced, and indel frequencies were quantified by TIDE software. The overall cutting efficiency for each gene is between 35 and 45%, meaning that over 35% of the cells carry an indel (insertion or deletion) in the targeted gene leading to a deletion, insertion or a frameshift mutation in the reading frame, thereby rendering the gene of interest nonfunctional.

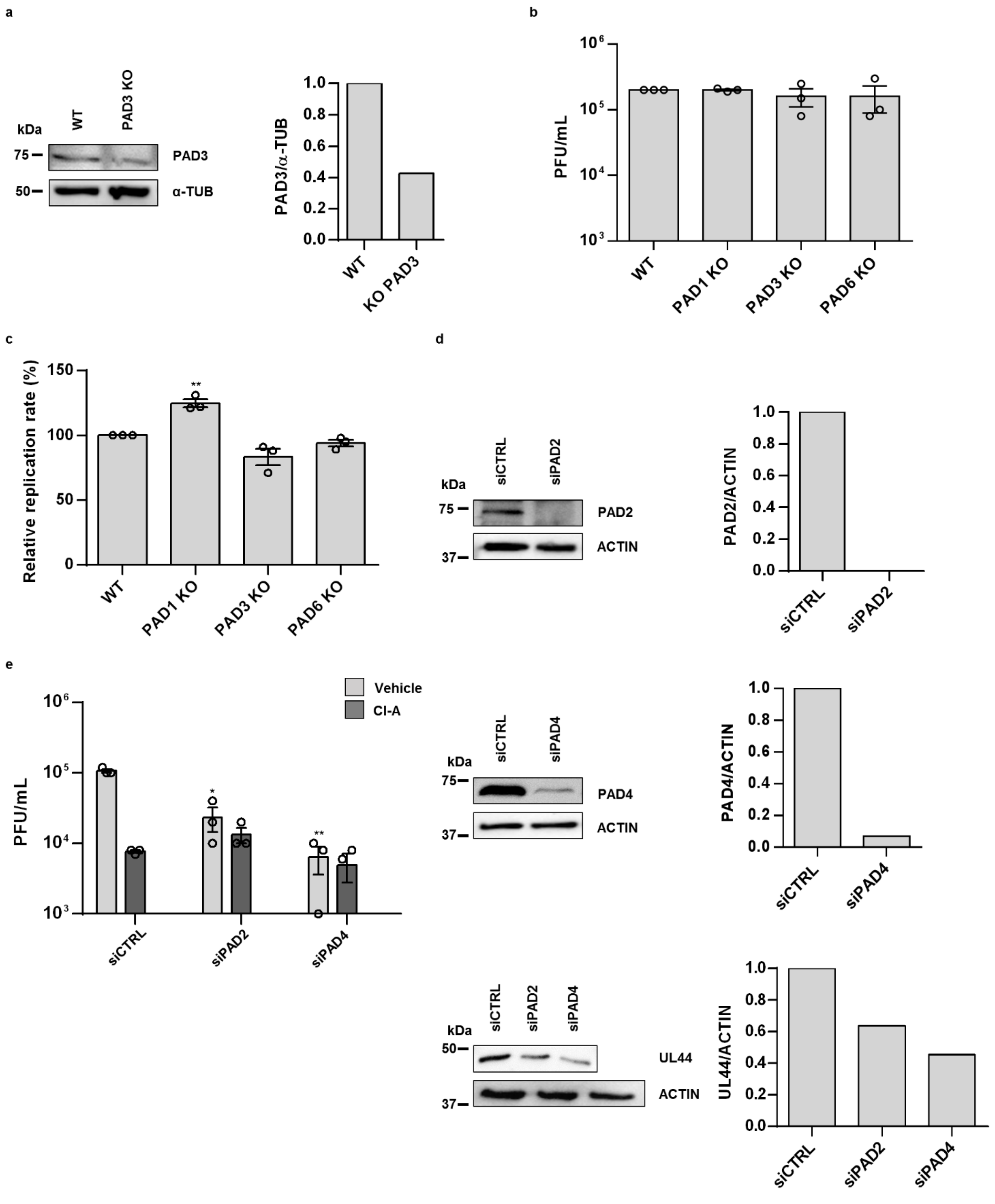

Supplementary Fig. 4. Effect of PAD1, PAD3 and PAD6 gene knockout on HCMV replication. **a,** Knockout (KO) gene variants in HFFs for PAD1 (PAD1 KO), PAD3 (PAD3 KO) and PAD6 (PAD6 KO) were generated using CRISPR/Cas9 technology. The efficiency of PAD3 protein depletion was verified by immunoblotting using an antibody against PAD3 and an anti-α-tubulin (α-

TUB) antibody as loading control. The Western blot and densitometric analysis are representative of three independent experiments. **b,** HFFs depleted of PAD1, PAD3, PAD6 were infected with HCMV at an MOI of 0.1. The extent of virus replication was measured at the indicated time points by standard plaque assay. Values are expressed as means ± SEM (error bars) of three independent experiments (one-way ANOVA followed by Bonferroni's post test). **c,** To determine the number of viral DNA genomes in HCMV-infected HFFs PAD1, PAD3, PAD6 KO cells (MOI 0.1), viral DNA was isolated at 144 hpi and analyzed by qPCR, using primers amplifying a segment of the IE1 gene. GAPDH was used to normalize HCMV genome counts. Values are expressed as mean ± SEM (error bars) of three independent experiments (Mock *vs.* PAD1 KO P=0.00966, one-way ANOVA followed by Bonferroni's post test). **d,** HFFs were transiently electroporated with specific siRNA against PAD2 and PAD4 (siPAD2, siPAD4) or control (siCTRL). After 24 h, transfected cells were Mock-infected or infected with HCMV at an MOI of 1. At 48 hpi, cells were harvested, and the efficiency of PAD2 and PAD4 protein silencing was determined by Western blot analysis using antibodies against PAD2, PAD4, UL44 and ACTIN as loading control. The blot shown along with its densitometric analysis is representative of three independent experiments. Values are expressed as fold change in PAD2, PAD4 and UL44 expression normalized to ACTIN. **e,** HFFs depleted of PAD2 and PAD4 by siRNA transfection were infected with HCMV at an MOI of 0.1 and treated or not with 100 µM Cl-A. The extent of virus replication was measured at 144 hpi by standard plaque assay. Values are expressed as means ± SEM (error bars) of three independent experiments (Vehicle: siCTRL *vs.* siPAD2 P< 0.05, Vehicle: siCTRL *vs.* siPAD4 P< 0.01; two-way ANOVA followed by Bonferroni's post-tests). Data are shown as the mean ± SEM, *P < 0.05, **P < 0.01, ***P < 0.001.

.

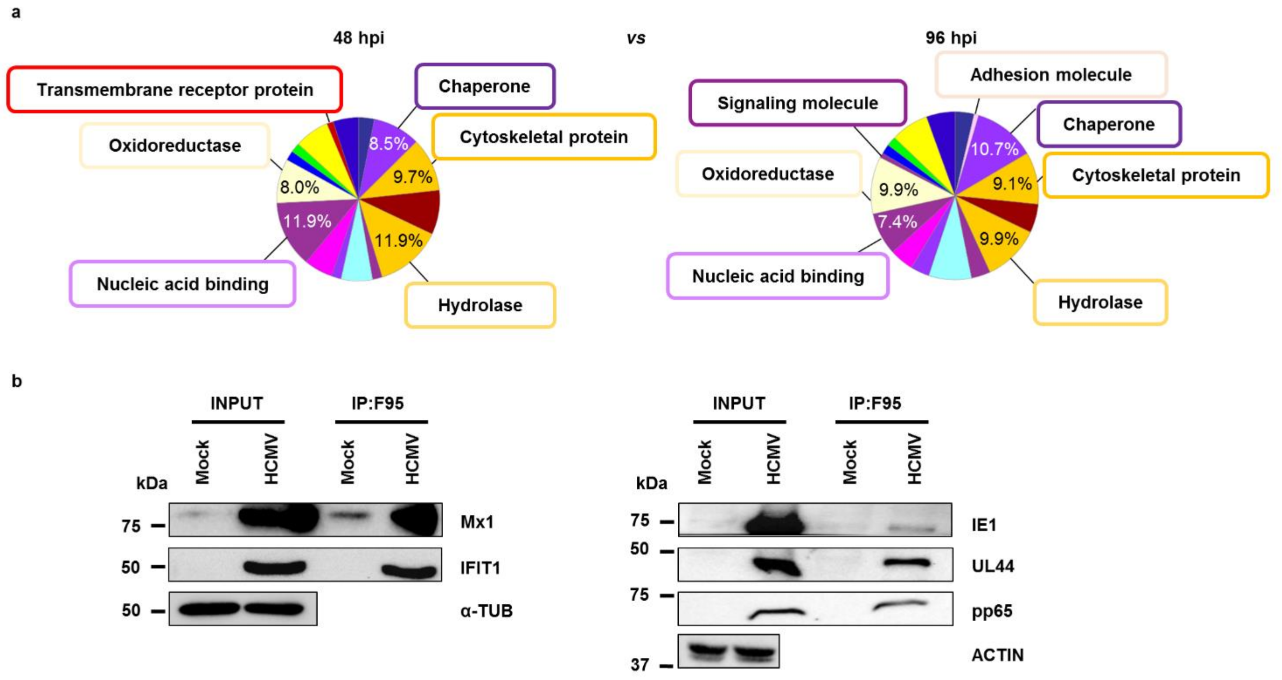

Supplementary Fig. 5. HCMV infection triggers citrullination of IFIT1, Mx1 and HSPH1 proteins. a, The pie charts show the classification of citrullinated proteins at 48 hpi (left) and 96 hpi (right) based on protein classes. **b,** Immunoprecipitation (IP) of total cell extracts (INPUT) from Mock and infected HFFs at 48 hpi using the anti-peptidylcitrulline antibody F95. The IP complexes were analyzed by Western blotting using antibodies against Mx1, IFIT1 (left panel), IEA (recognizing IE1-72- and IE2-86 kDa), UL44 and pp65 (right panel). Equal loading was assessed by α-tubulin (α-TUB) or ACTIN immunoblotting. The blot shown is representative of three independent experiments.

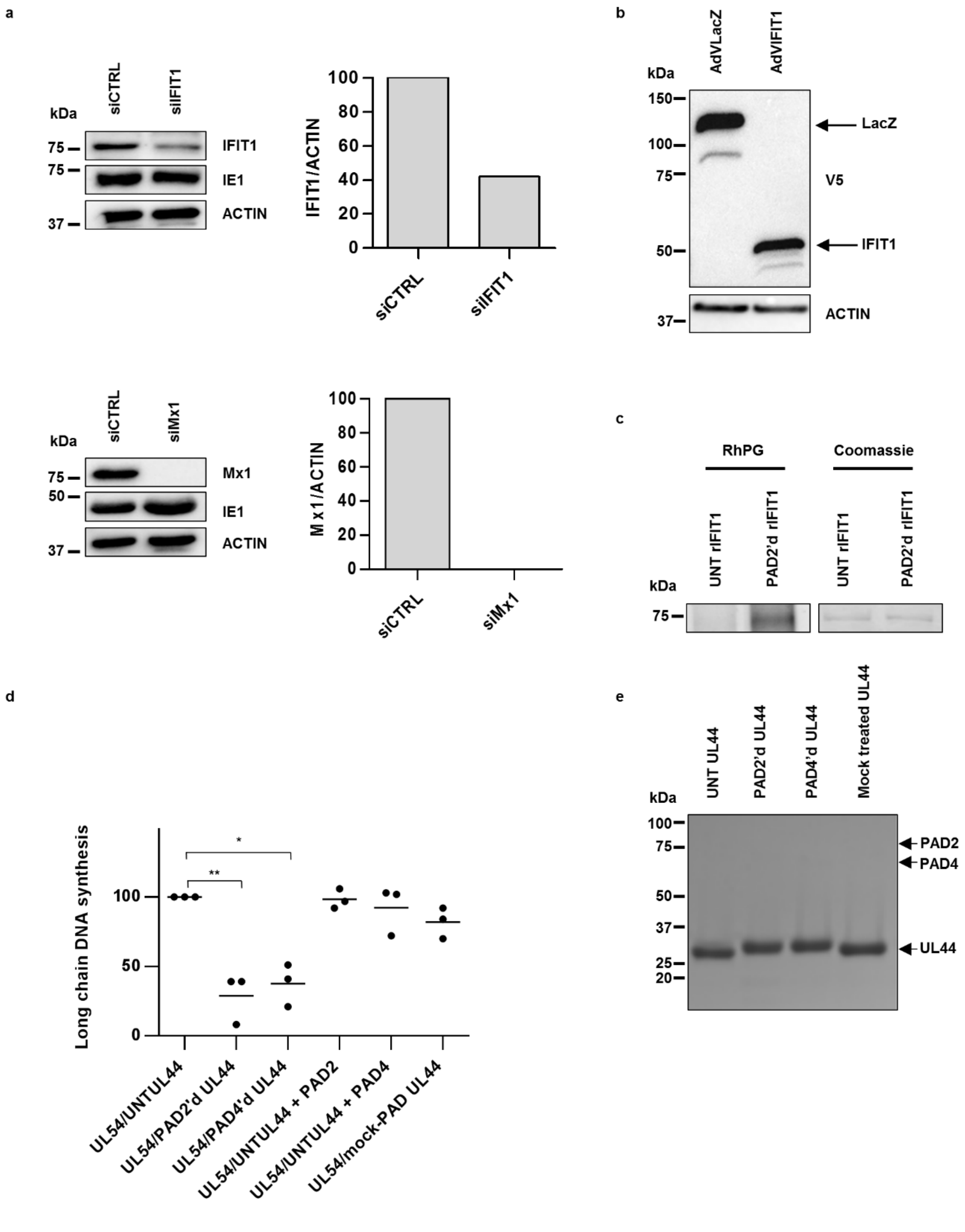

Supplementary Fig. 6. Silencing and overexpression of IFIT1 and Mx1. a, HFFs were transiently electroporated with specific siRNA against IFIT1 or Mx1 (siIFIT1, siMx1) or control (siCTRL). After 24 h, transfected cells were Mock-infected or infected with HCMV at an MOI of 1. At 48 hpi, cells were harvested, and the efficiency of IFIT1 and Mx1 protein silencing was assayed by Western blot analysis using antibodies against IFIT1, Mx1, IEA (recognizing IE1-72 and IE2-86 kDa), and ACTIN as loading control. The blot shown along with its densitometric analysis is representative of three

independent experiments. Values are expressed as fold change in IFIT1 and Mx1 expression normalized to ACTIN. **b,** HFFs were infected with AdVIFIT1V5 or AdVLacZV5 at an MOI of 10. At 72 hpi, cells were harvested, and the efficiency of IFIT1 and LacZ overexpression was determined by Western blot analysis using antibodies against the V5 tag or ACTIN as loading control. The blot shown along with its densitometric analysis is representative of three independent experiments. Values are expressed as fold change in IFIT1 expression normalized to ACTIN. **c,** Recombinant IFIT1 (3 μg) was left untreated (UNT rIFIT1) or citrullinated *in vitro* with PAD2 (PAD2'd rIFIT1), then exposed to an Rh-PG citrulline-specific probe (left panel) and subjected to gel electrophoresis to detect citrullinated proteins. Equal loading was assessed by Coomassie blue staining (right panel). One representative gel of three independent experiments is shown. **d, Effects of citrullination on UL44.** Three replicate long-chain DNA synthesis experiments were performed, quantified, and normalized to the values of UNT controls in each replicate as described in the Methods (UL54/UNTUL44 *vs.* UL54/PAD2'd UL44 P=0.0041, UL54/UNTUL44 *vs.* UL54/PAD4'd UL44 P=0.00293, one-way ANOVA with Dunnett's correction in GraphPad Prism version 8.4.3 for Windows. **e,** 2.2 μg of untreated UL44ΔC290 (lane 1), or PAD2 treated (lane 2), PAD4 treated (lane 3), or mock treated (lane 4) UL44ΔC290 purified from citrullination reactions were analysed by SDS-PAGE and Coomassie blue staining alongside molecular weight markers. Faint bands that can be observed migrating similarly to the 75 kDa marker correspond to trace amounts of residual PAD2 or PAD4 in lanes 2 and 3. One representative blot of three independent experiments is shown. Data are shown as the mean ± SEM, *P < 0.05, **P < 0.01, ***P < 0.001.

SUPPLEMENTARY TABLE

Supplementary Table 1. Oligonucleotide primer sequences for qPCR

GENE OF INTEREST	SEQUENCE (5'-3')
**PADI1 FW**	TCCAGAGACCCTGAAGCTGT
**PADI1 RV**	GTGCAGCTGTCCCTGAAGAT
**PADI2 FW**	ACCTCCTCAGCCTCCCC
**PADI2 RV**	CCTACCTCTGGACCGATGTC
**PADI3 FW**	GCGTCCCATAGACCTCAAAC
**PADI3 RV**	CAGAGAATCGTGCGTGTGTC
**PADI4 FW**	CCTGTGGATTTCTTCTTGGC
**PADI4 RV**	GGGCACCTTGACTCAGCTT
**PADI6 FW**	CAAGGTATAGGCGTGCTGGT
**PADI6 RV**	TCCTCCATACCTCCAAGGAA
**IE1 FW**	TCAGTGCTCCCTGATGAGA
**IE1 RV**	GATCAATGTGCGTGAGCACC

Supplementary Table 2. Primary antibodies for Western blotting.

ANTIBODY	Cat number	DILUTION	SOURCE
Anti-peptidyl-citrulline, clone F95	MABN328	1:500	Sigma Aldrich, Milan, Italy
Anti-PAD2	SML-ROI002-EX	1:1500	Cosmo Bio, Eboli, Italy
Anti-IFIT1	PA3-848	1:1000	ThermoFischer, Waltham, USA
Anti-MX1	ab95926	1:500	Abcam, Cambridge, United Kingdom
Anti-PAD6	ab169416	1:500	Abcam, Cambridge, United Kingdom
Anti-PAD1	ab24008	1:1000	Abcam, Cambridge, United Kingdom
Anti-PAD3	ab172959	1:500	Abcam, Cambridge, United Kingdom
Anti-PAD4	ab128086	1:500	Abcam, Cambridge, United Kingdom
Anti-alpha-tubulin	39527	1:1000	Active Motif, La Hulpe, Belgium
Anti-actin clone C4	MAB1501	1:1000	Sigma Aldrich, Milan, Italy
Anti-IEA	P1215	1:1000	Virusys Taneytown, USA
Anti-UL44	P1202	1:1000	Virusys Taneytown, USA
Anti-pp28	P1207	1:1000	Virusys Taneytown, USA

Anti-pp65	CA003	1:1000	Virusys Taneytown, USA
Anti-V5	R960-25	1:1000	ThermoFischer, Waltham, USA
Anti-Histone H3 (citrulline R2+R8+R17)	Ab5103	1:2000	Abcam, Cambridge, United Kingdom

Supplementary Table 3. Primers used to prepare the *PADI2* and *PADI4* promoter reporter plasmids

NAME	SEQUENCE (5’-3’)	POSITION
**PADI4 Promoter FW**	5’-TAACTCGAGGGCTCTATGAAGGCAGGA-3’	-1977/-1960
**PADI2 Promoter FW**	5’-TATCTCGAGCCCACACTGATTGTCATC-3’	-868/-850
**PAD4 Promoter RV**	5’- TAGAAGCTTCGTCGGGCTAGCTCGTC-3’	+27/+10
**PAD2 Promoter RV**	5’-TATAAGCTTCCTCCCCGCCGCAGTGC-3’	+63/+80

Position number +1 corresponding to the first nucleotide of the transcription initiation site.

Supplementary Table 4. CRISPR guide RNAs

GENE OF INTEREST	gRNA TARGET SEQUENCE
**PADI1 #1**	gCATCAGTGTCTAGCGGCCAA
**PADI2 #1**	gACCTCTGGACCGATGTCTAC
**PADI3 #1**	gTTACCCATAAATGTCCACGA
**PADI4 #1**	gCAATATCCACGACCACCCCT
**PADI6 #1**	gCAGGTGGATGATACTCTGGA

Supplementary Table 5. Primers for TIDE analysis

GENE OF INTEREST	SEQUENCE (5’-3’)
**PADI1 FW**	GGGGGTGTCCAAGGGTTTAC
**PADI1 RV**	TGGAGGTTTGCGTGTTTTGG
**PADI2 FW**	GATGACTTCGCCACGACCT
**PADI2 RV**	CGGAGGGTTACGGGTGAAAG
**PADI3 FW**	CACAGCTAAGTCCAACACCAG
**PADI3 RV**	CTAGGCTCATAGGCTCAGGATG

**PADI4 FW**	AGTAAGCACTGGCTGCTGTTT
**PADI4 RV**	ATCACGAGCTCTTCCACAGG
**PADI6 FW**	CCCCTTCTTGGGAATTGGGTG
**PADI6 RV**	GCAAAACAACGTGTTTCCCAG

Supplementary Table 6. Primers for AdV production

NAME	SEQUENCE (5'-3')
**AdVIFIT1V5 FW**	AACCGTCAGATCGCCTGGAGACGCCATCCACGCTGTTTTGACCTCCATAGAAGACACCGGGACCGATCCAGCCTGGATCCATGAGTACAAATGGTGATGA
**AdVIFIT1V5 RV**	TATAGAGTATACAATAGTGACGTGGGATCCCTACGTAGAATCAAGACCTAGGAGCGGGTTAGGGATTGGCTTACCAGCGCTAGGACCTTGTCTCACAGAGT
**AdVLacZV5 FW**	AACCGTCAGATCGCCTGGAGACGCCATCCACGCTGTTTTGACCTCCATAGAAGACACCGGGACCGATCCAGCCTGGATCCATGATAGATCCCGTCGTTTT
**AdVLacZV5 RV**	TATAGAGTATACAATAGTGACGTGGGATCCCTACGTAGAATCAAGACCTAGGAGCGGGTTAGGGATTGGCTTACCAGCGCTTTTTTGACACCAGACCAACT
**Sequencing AdV CMV promoter**	AATGTCGTAACAACTCCG
**Sequencing AdV CMV PolyA**	ACCTGATGGTGATAAGAAG
