# Supplementary material for: Human cytomegalovirus-induced host protein citrullination is crucial for viral replication
Source: Nat Commun. 2021 Jun 23;12:3910. doi: 10.1038/s41467-021-24178-6 (PMC8222335; doi:10.1038/s41467-021-24178-6)
Supplement: Supplementary file 2 — Description of Additional Supplementary Files [file 41467_2021_24178_MOESM2_ESM.pdf]

Description of Additional Supplementary Files

File Name: Supplementary Data 1
Description: **Host Cell and Viral Citrullinome.**
1. **Host and Viral Spectral Counting (SC).** Enrichment ratios of host and viral citrullinated proteins identified from HCMV vs. mock-infected HFFs at 48 and 96 hpi. The values for three independent experiments are shown. **ID. Protein. Unique_pep_SC (hpi 48, and 96)**
2. **48h Post Infection.** Host and viral citrullinated proteins identified from HCMV vs. mock-infected HFFs at 48 hpi. Average and P-value are calculated from three independent experiments**. ID. Protein. Average 48 hpi SC and Mock SC. P-value. Ratio 48 hpi/Mock48.**
3. **96h Post Infection.** Host and viral citrullinated proteins identified from HCMV vs mock-infected HFFs at 96 hpi. Average and P-value are calculated from three independent experiments. **ID. Protein. Average 96 hpi SC and Mock SC. P-value. Ratio 96 hpi/Mock96.**
4. **Time course comparison.** Host and viral citrullinated proteins identified from HCMV vs. mock-infected HFFs at 48 and 96 hpi. Average and P-value are calculated from three independent experiments. **ID. Protein. Average 96 hpi SC and 48 hpi SC. P-value. Ratio 48 hpi/96 hpi.**

File Name: Supplementary Data 2
Description: **Host Cell citrullinome.**
1. **Host Cell Spectra Counting.** Enrichment ratios of host citrullinated proteins identified from HCMV vs. mock-infected HFFs at 48 and 96 hpi. Average and P-value are calculated from three independent experiments. **ID. Protein. Unique_pep_SC (hpi 48, and 96)**
2. **48 h Post Infection.** Host citrullinated proteins identified from HCMV vs. mock-infected HFFs at 48 hpi. Average (avg) and P-value are calculated from three independent experiments. **ID. Protein. Mock 48 avg SC. 48 hpi avg SC. P-value. Ratio 48 hpi/Mock48**
3. **96 h Post Infection.** Host citrullinated proteins identified from HCMV vs. mock-infected HFFs at 96 hpi. Average and P-value are calculated from three independent experiments. **ID. Protein. Mock 96 avg SC. 96hpi avg SC. P-value. Ratio 96 hpi/Mock96.**
4. **Time course comparison.** Host citrullinated proteins identified from HCMV vs. mock-infected HFFs at 48 and 96 hpi. Average and P-value are calculated from three independent experiments. **ID. Protein. 48 avg and 96 avg. P-value. Ratio 48 hpi/96 hpi.**

File Name: Supplementary Data 3
Description: **Viral citrullinome.**
1. **Viral Spectra Counting.** Enrichment ratios of viral citrullinated proteins identified from HCMV vs. mock-infected HFFs at 48 and 96 hpi. Average and P-value are calculated from three independent experiments **ID. Protein. Unique_pep_SC (hpi 48 and 96).**
2. **48 h Post Infection.** Viral citrullinated proteins identified from HCMV vs. mock-infected HFFs at 48 hpi. Average and P-value are calculated from three independent experiments. **ID. Protein. 48 hpi avg SC. Mock48 avg SC. P-value. Ratio 48 hpi/Mock48.**
3. **96 h Post Infection.** Viral citrullinated proteins identified from HCMV vs. mock-infected HFFs at 96 hpi. Average and P-value are calculated from three independent experiments. **ID. Protein. 96 hpi avg SC and Mock96 avg SC. P-value. Ratio 96 hpi/Mock96**

File Name: Supplementary Data 4
Description: **Host Cell citrullinome upon wtAD169 infection.**
1. Host citrullinated proteins identified from HCMV wtAD169- vs mockinfected HFFs at 48 hpi**. Identified proteins. Accession Number. Molecular weight. Log2[Fold change, Medium/Light]*. T-test (pvalue).**

File Name: Supplementary Data 5
Description: **Host Cell citrullinome upon wtAD169-deltaIE1 infection.**
Host citrullinated proteins identified from HCMV AD169-ΔIE1 vs mock-infected HFFs at 48 hpi. **Identified proteins. Accession Number. Molecular weight. Log2[Fold change, Medium/Light]*. Ttest (p-value).**

File Name: Supplementary Data 6
Description: **modifiedPeptidesIFIT1.**
IFIT1 peptides identified by LC -MS/MS analysis of recombinant IFIT1, mock - or PAD2 -treated, and their modifications, as extracted from the modificationSpecificPeptides.txt output file of MaxQuant. **Column name:** relative description: **Sequence:** The identified AA sequence of the peptide; **Modifications:** Post -translational modifications contained within the sequence. When no modifications exist, this is set to 'unmodified'; **Mass (Da):** Charge corrected mass of the precursor ion; **Proteins:** The identifiers of the proteins this particular peptide is associated with; **Gene Names:** Names of genes this peptide is associated with.; **Position of cleavage:** Position of the last amino acid of this peptide in the protein sequence. (one -based); **Missed cleavages:** Number of missed enzymatic cleavages.; **Position of citrulinated site**: Position of the site in the respective protein sequence. The index of the first amino acid in the sequence is 1.; **Citrullination number**: Number of Citrullination on this peptide.; **Identification type IFIT1+PAD2**: Indicates whether this experiment was identified by MS/MS or only by matching between runs.; **Identification type IFIT1 mock:** Indicates whether this experiment was identified by MS/MS or only by matching between runs.; **Charges:** All charge states that have been observed on this peptide.; **PEP:** Posterior Error Probability of the identification. This value essentially operates as a p - value, where smaller is more significant.; **Score:** Andromeda score for the best identified among the associated MS/MS spectra. **Intensity IFIT1+PAD2:** Summed up eXtracted Ion Current (XIC) of all isotopic clusters associated with the identified AA sequence. In case of a labeled experiment this is the total intensity of all the isotopic patterns in the label cluster.; **Intensity IFIT1 mock:** Summed up eXtracted Ion Current (XIC) of all isotopic clusters associated with the identified AA sequence. In case of a labeled experiment this is the total intensity of all the isotopic patterns in the label cluster.
