# Supplementary material for: Human cytomegalovirus-induced host protein citrullination is crucial for viral replication
Source: Nat Commun. 2021 Jun 23;12:3910. doi: 10.1038/s41467-021-24178-6 (PMC8222335; doi:10.1038/s41467-021-24178-6)
Supplement: Supplementary file 9 — Reporting Summary [file 41467_2021_24178_MOESM9_ESM.pdf]

nature research

Corresponding author(s): Santo Landolfo
Last updated by author(s): May 14, 2021

Reporting Summary

Nature Research wishes to improve the reproducibility of the work that we publish. This form provides structure for consistency and transparency in reporting. For further information on Nature Research policies, see our Editorial Policies and the Editorial Policy Checklist.

Statistics

For all statistical analyses, confirm that the following items are present in the figure legend, table legend, main text, or Methods section.

n/a	Confirmed	
☐	☒	The exact sample size (*n*) for each experimental group/condition, given as a discrete number and unit of measurement
☐	☒	A statement on whether measurements were taken from distinct samples or whether the same sample was measured repeatedly
☐	☒	The statistical test(s) used AND whether they are one- or two-sided<br>*Only common tests should be described solely by name; describe more complex techniques in the Methods section.*
☐	☒	A description of all covariates tested
☐	☒	A description of any assumptions or corrections, such as tests of normality and adjustment for multiple comparisons
☐	☒	A full description of the statistical parameters including central tendency (e.g. means) or other basic estimates (e.g. regression coefficient) AND variation (e.g. standard deviation) or associated estimates of uncertainty (e.g. confidence intervals)
☐	☒	For null hypothesis testing, the test statistic (e.g. *F*, *t*, *r*) with confidence intervals, effect sizes, degrees of freedom and *P* value noted<br>*Give P values as exact values whenever suitable.*
☒	☐	For Bayesian analysis, information on the choice of priors and Markov chain Monte Carlo settings
☒	☐	For hierarchical and complex designs, identification of the appropriate level for tests and full reporting of outcomes
☒	☐	Estimates of effect sizes (e.g. Cohen's *d*, Pearson's *r*), indicating how they were calculated

Our web collection on statistics for biologists contains articles on many of the points above.

Software and code

Policy information about availability of computer code

Data collection	Microsoft Excel 2016, Typhoon scanner (Amerhsam), ChemiDoc MP Imaging system (Biorad), VICTOR3 Multilabel Reader (PerkinElmer), Real time Mx3000P apparatus (Stratagene), LTQ-Orbitrap Discovery mass spectrometer (ThermoFisher) coupled to an Easy-nLC HPLC (Thermo Fisher), LC-MS/MS, using an EASY-nLC 1200 system (Thermo Fisher Scientific) coupled to a Q-Exactive HF mass spectrometer (Thermo Fisher Scientific), NanoAcquity UPLC (Waters Corporan), Orbitrap Fusion Lumos Tribrid (Thermo Fisher Scientific), XCalibur software (v.3.0 DDA), Wallac 1420 work station.
Data analysis	GraphPad Prism 5.0 and 8.4.3, ImageLab 6.1, Microsoft Excel 2016, TIDE version 1.0.2, MAxQuant version 1.6.0.15, SEQUEST output files were filtered using DTASelect 2.0, Maxquant 1.6.14 and its integrated Andromeda search engine using the Swiss-Prot human and Uniprot HCMV, PANTHER 16.0, Peptide identification and quantification by MS: MaxQuant (v1.6.0.15 https://maxquant.org), Chromas 2.6.6.

For manuscripts utilizing custom algorithms or software that are central to the research but not yet described in published literature, software must be made available to editors and reviewers. We strongly encourage code deposition in a community repository (e.g. GitHub). See the Nature Research guidelines for submitting code & software for further information.

Data

Policy information about availability of data

All manuscripts must include a data availability statement. This statement should provide the following information, where applicable:

- Accession codes, unique identifiers, or web links for publicly available datasets
- A list of figures that have associated raw data
- A description of any restrictions on data availability

Source data are provided with this paper. The mass spectrometry proteomics data have been deposited to the ProteomeXchange Consortium via the PRIDE [1]

partner repository with the dataset identifier PXD025803, and PXD025818.

Field-specific reporting

Please select the one below that is the best fit for your research. If you are not sure, read the appropriate sections before making your selection.

☒ Life sciences ☐ Behavioural & social sciences ☐ Ecological, evolutionary & environmental sciences

For a reference copy of the document with all sections, see nature.com/documents/nr-reporting-summary-flat.pdf

Life sciences study design

All studies must disclose on these points even when the disclosure is negative.

Sample size	Because the variation in the assays used is small and we are interested in large effects, the sample sizes used, as indicated in the manuscript, were deemed appropriate.
Data exclusions	Data was generally not excluded from the analysis.
Replication	All attemps at replication were successful. The experiments were performed independently 3 times.
Randomization	Randomization was not used since these are no esperimental groups.
Blinding	Blinding was performed with antiviral assays. In vitro experiments were not blind because some of the results are quantitative, or not subjective. Blinding was not feasible in these experiments.

Reporting for specific materials, systems and methods

We require information from authors about some types of materials, experimental systems and methods used in many studies. Here, indicate whether each material, system or method listed is relevant to your study. If you are not sure if a list item applies to your research, read the appropriate section before selecting a response.

Materials & experimental systems

n/a	Involved in the study	
☐	☒	Antibodies
☐	☒	Eukaryotic cell lines
☒	☐	Palaeontology and archaeology
☒	☐	Animals and other organisms
☒	☐	Human research participants
☒	☐	Clinical data
☒	☐	Dual use research of concern

Methods

n/a	Involved in the study	
☒	☐	ChIP-seq
☒	☐	Flow cytometry
☒	☐	MRI-based neuroimaging

Antibodies

Antibodies used

Sigma-Aldrich anti-peptidyl-citrulline, clone F95, CAT MABN 328, LOT2918925-2861323;
Cosmo Bio anti-PAD2 CAT SML-ROI002-EX LOT 1707;
Thermo fisher anti-IFIT1 CAT PA 3-848 LOT VB 300277;
Thermo fisher anti-V5 CAT R960-25 LOT 2106326
Virusys anti-pp65 CAT CA003-100 LOT B1653064;K1448061;
Virusys anti-pp28 CAT p1207 LOT D1652086;
Virusys anti-UL44 CAT P1202 LOT11242136
ABCAM anti-Mx1 CAT 95926 LOTGR3180562-18;
Virusys anti-IEA CAT P1215 LOT. 1450068 ;
ABCAM anti-PAD6 CAT ab169416 LOT GR3174350-1;
ABCAM anti-PAD4 CAT ab128086 LOT GR193654-33; LOTGR3266397-7
ABCAM anti-PAD3 CAT ab172959 LOT EPR12165(B);
ABCAM anti-PAD1 CAT ab24008 LOT GR307572-4;
Sigma-Aldrich anti-actin clone C4 CAT MAB1501 LOT 3423208;
Active Motif anti-alpha-tubulin (mAb), clone 5-B-1-2 cat 39527 LOT 01008001;
Amersham anti rabbit IgG, HRP-linked species -specific whole antibody (from donkey) CAT NA934;
Amersham ECL Mouse IgG, HRP-linked whole Ab (from sheep) CAT NXA931-1ML;
ABCAM Anti-Histone H3 (citrulline R2 + R8 + R17) CAT ab5103 LOT GR314058-2;
Sigma-Aldrich Goat anti-Mouse IgM Antibody, µ chain, HRP conjugate CAT AP128P LOT12-489.

Validation

Validation was provided by the vendor for all antibodies used in this study.
Technical specfication from the vendor website:
CLONE F95: This Anti-peptidyl-citrulline, clone F95 Antibody is validated for use in Immunohistochemistry and Western Blotting and Immunocytochemistry for the detection of peptidyl-citrulline.
Anti-PAD2: Tested applications :• Immunofluorescence (1:1001:300 dilution)• immunohistochemistry (1:1001:300 dilution)•Western Blot (1:5001:2,000 dilution).
Anti-IFIT1-Thermo fisher:PA3-848 has been successfully used in Western blot and ICC/IF applications to detect a 39-58 kDa band.
Anti V5: recognizes amino acid sequence: -Gly-Lys-Pro-Ile-Pro-Asn-Pro-Leu-Leu-Gly-Leu-Asp-Ser-Thr-.This antibody is functionally tested against 20 ng of an E. coli expressed fusion protein containing a V5 epitope using a chemiluminescent substrate at a 1 minute exposure. This antibody has also been tested in Western blot against 25 ng of recombinant Positope protein.
Anti pp65: Western Blot validation with Antigen Virusys # CV001 CMV (Infected Cell Extract at 10 µg/cm).
Anti pp28: Western Blot validation with Antigen Virusys # CV001 CMV (Infected Cell Extract at 10 µg/cm).
Anti Ul44: Western Blot validation with Antigen Virusys # CV001 CMV (Infected Cell Extract at 10 µg/cm).
Anti IEA: Western Blot validation with Antigen Virusys # CV001 CMV (Infected Cell Extract at 10 µg/cm).
Anti MX1: Our Ab promise guarantee covers the use of ab95926 in the following tested applications:WB.
Anti PAD6: Our Ab promise guarantee covers the use of ab16480 in the following tested applications:WB, IHC-P.
Anti PAD4: Our Ab promise guarantee covers the use of ab16480 in the following tested applications:WB, IHC-P.
Anti PAD3: Our Ab promise guarantee covers the use of ab16480 in the following tested applications:WB, IP.
Anti PAD1: Our Ab promise guarantee covers the use of ab16480 in the following tested applications:WB, IHC-P.
Anti actin: Routinely evaluated by Western Blot on A431 lysates.
Anti tubulin: Applications Validated by Active Motif: WB, ICC/IF.

Eukaryotic cell lines

Policy information about cell lines

Cell line source(s)

Human foreskin fibroblasts (HFFs, ATCC CAT SCRC-1041™), African green monkey kidney cells (Vero, SIGMA CAT 84113001), 293 Cell Line human from human kidney (embryonic HEK 293) CAT 85120602, CD4+ lymphoblastoid T (C8166, ECACC CAT 88051601), 293T (HEK293T, ATCC® CAT CRL-3216™).

Authentication

HFF: The cell line was established by ATCC in 2003 from normal human foreskin pooled from two individuals. Organism: Homo sapiens, human; Tissue: Skin (foreskin) Disease: Normal Cell Type; Fibroblast Age: Newborn; Gender: male Morphology: fibroblast; Growth Properties: Adherent.
VERO: The cell line was established by SIGMA from the kidney of a normal adult African Green monkey. Biological source: African green monkey kidney; Growth mode: Adherent; karyotype: 2n = 60, modal no. 58; Morphology : Fibroblast-like.
293: The cell line was established by SIGMA from human kidney (embryonic). karyotype 2n 46, hypotriploid, modal no. 64 STR-PCR Data: Amelogenin: X CSF1PO: 11,12 D13S317: 12,14 D16S539:9,13 D5S818: 8,9 D7S820: 11,12 THO1: 7,9.3 TPOX: 11 vWA: 16,19.
C8166: Clone of C63/CRII-4 derived by fusion of primary umbilical cord blood cells with HTLV-1 producing line from adult T cell leukaemia lymphoma patient. Contain defective HTLV-1 genome.
293T: cell line is a highly transfectable derivative of human embryonic kidney 293 cells, and contains the SV40 T-antigen. (originally referred as 293tsA1609neo). STR Profile CSF1PO: 11,12 D13S317: 12,14 D16S539: 9,13 D5S818: 8,9 D7S820: 11 TH01: 7, 9.3 TPOX: 11 vWA: 16,19 Amelogenin: X.

Mycoplasma contamination

We confirm that all cell lines tested negative for mycoplasma contamination.

Commonly misidentified lines
(See ICLAC register)

No commonly misidentified cell lines were used in the study.
